# In Silico Structural Analysis of Human β‐Glucuronidase for Antibody–Drug Conjugates Optimization

**DOI:** 10.1002/prot.70077

**Published:** 2025-10-31

**Authors:** Giorgia Canini, Simona Saporiti, Crescenzo Coppa, Mara Rossi, Fabio Centola, Alessandro Arcovito

**Affiliations:** ^1^ Fondazione Policlinico Universitario “A. Gemelli” Roma Italy; ^2^ Analytical Excellence and Program Management Merck Serono S.P.A Rome Italy; ^3^ Dipartimento di Scienze Biotecnologiche di Base, Cliniche Intensivologiche e Perioperatorie Università Cattolica del Sacro Cuore Roma Italy

**Keywords:** antibody–drug conjugates, computational study, human β‐glucuronidase, molecular dynamics

## Abstract

Human β‐glucuronidase (HGUSB), a key lysosomal glycosyl hydrolase for the degradation pathway of glycosaminoglycans (GAGs), plays a crucial role in cell proliferation and inflammation, making it a promising target for novel therapeutic strategies including antibody‐drug conjugates (ADCs) with β‐glucuronic linkers. In this study, molecular docking and molecular dynamics (MD) simulations were performed to investigate the conformational stability of HGUSB in complex with different ligands, including substrates, inhibitors, and β‐glucuronic linkers. Our rationale approach includes the evaluation of commercial substrates and a known inhibitor with different binding stoichiometries to identify the most favorable configuration and the most stable conformation of the enzyme. Based on the binding mechanism of HGUSB to these well‐known ligands, the interaction with commercial linkers was evaluated, providing a structural determination of the recognition mechanism between the enzyme and ADCs. MD simulations on HGUSB::Linker complexes revealed that the maleimide‐containing hydrophilic β‐glucuronide, exhibited the most stable binding making it the best fitting linker among those analyzed in this study. Overall, this study identifies the optimal binding configuration of the HGUSB enzyme for investigating small molecule interactions and, despite the endogenous homotetrameric assembly, justifies the use of a simplified monomeric model for the study of larger macromolecular complexes, like linker analysis, ensuring an efficient and accurate computational approach. These findings lay the groundwork for a rationale optimization of β‐glucuronic linker‐based ADCs, offering new perspectives for targeted cancer therapies.

## 1 Introduction

Glycosaminoglycans (GAGs) are linear polysaccharides characterized by repeating disaccharide units, an amino sugar (which can be N‐acetylglucosamine or N‐acetylgalactosamine) and a uronic acid (which can be either glucuronic acid or iduronic acid) [1, 2]. Degradation of GAGs inside lysosomes mainly depends on the human lysosomal beta‐glucuronidase (HGUSB) enzyme which is encoded by the *HGUSB* gene on chromosome 7 and belongs to the glycoside hydrolase (GH) family 2. This enzyme catalyzes the hydrolysis of glucuronic acid of GAGs (like dermatan sulfate, heparan sulfate, and chondroitin sulfate), playing a central role in cellular homeostasis [3].

**FIGURE 1:**
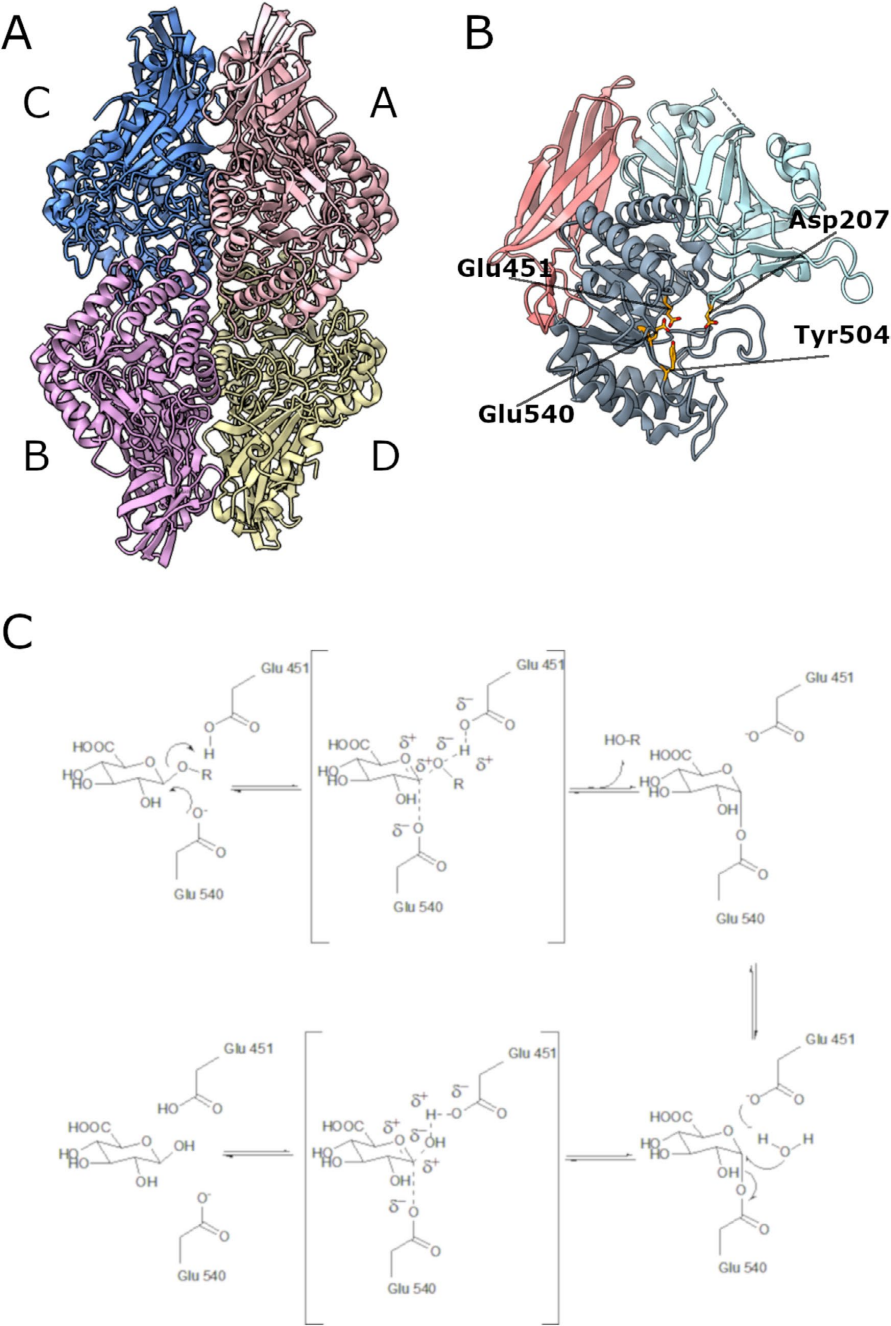
HGUSB structure and reaction mechanism. (A) X‐ray structure of HGUSB in its tetrameric form. Distinct colors and letters are used to discern the monomers. (B) Representation of the three structural domains in the HGUSB monomer. The barrel‐like shape domain is shown in cyan, the Ig‐like domain is shown in pink, and the C‐terminal domain is shown in gray. The key amino acids of the catalytic site are reported in orange. (C) Reaction retaining mechanism for HGUSB, adapted from Yang Y. et al. and Alexander W. Wong et al. [14, 15].

HGUSB also plays an important role outside of lysosomal degradation, particularly in cell proliferation and inflammation. Recent research on lysosomal function has uncovered its significant role in disease pathogenesis, paving the way for new therapeutic strategies [4]. HGUSB can be considered a physiologically important lysosomal glycosyl hydrolase with therapeutic potential as a biomarker in disease diagnosis, but also an endogenous bio‐activator in prodrug monotherapy, and enzyme replacement therapy [5]. In this context, the role of the enzyme in cell proliferation and inflammation also makes it a potential target for the development of anti‐cancer and anti‐inflammatory drugs [5, 6]. Specifically, in cancer therapy, the high activity of HGUSB in tumor cells [7, 8, 9, 10, 11] positions it as a promising target for Antibody Drug Conjugate (ADC)‐based treatments, facilitating the selective release of cytotoxic agents within malignant cells via cleavage of beta‐glucuronide linkers [12]. This targeted approach increases therapeutic efficacy while minimizing toxicity to normal tissues [13]. From a structural perspective, this intriguing enzyme is a constitutively glycosylated homotetramer, with each subunit composed of 651 amino acids for a total of 2604 residues (Figure 1A). Each monomer presents three structural domains as shown in Figure 1B. The first domain presents a barrel‐like shape with a jelly roll motif, the second domain displays an immunoglobulin‐like (Ig‐like) geometry, the third C‐terminal domain forms a TIM barrel motif [5, 16]. The third domain contains the active site of the HGUSB, which includes the following key residues: Glu451 (proton donor), Glu540 (carbonium ion stabilizer), Asp207 (same role as Glu540), and Tyr504 (unclear catalytic role) [16, 17]. HGUSB binds substrates containing glucuronic acid residues and deconjugates β‐d‐glucuronides to their corresponding aglycone and β‐d‐glucuronic acid through an SN2 reaction and “configuration retaining” mechanism, as reported in Figure 1C [5, 14, 18]. The initial phase of the reaction, when the substrate binds to the active site of the enzyme, involves the nucleophilic attack by one of the carboxylates on the sugar's anomeric center. Facilitated by general acid catalysis from the other carboxyl group, this process drives the departure of the aglycone, producing an enzymatic intermediate called α‐D‐glycosyl. Once the substrate is bound to the active site, a water molecule is typically activated by nearby residues, making it more reactive and promoting the hydrolytic cleavage of the intermediate by general base‐catalyzed action of water at the anomeric site. The covalent intermediate is formed, resulting in the cleavage of the glycosidic bond between the glucuronic acid residue and the rest of the substrate molecule. This process yields glucuronic acid and the remaining portion of the substrate. Both the formation and breakdown of the glycosyl‐enzyme intermediate occur through transition states characterized by oxocarbenium ion character. The products of the hydrolysis reaction, including free glucuronic acid and the cleaved portion of the substrate, are released from the active site of the enzyme [17].

According to this action mechanism (Figure 1C), HGUSB has recently emerged as a viable therapeutic target for drug delivery methods, exploiting its capacity to hydrolyze glucuronide prodrugs into active molecules [19, 20, 21].

Therefore, the aim of this study is to investigate the interaction mechanism between commercial β‐glucuronidase linkers and HGUSB thus providing structural insights useful for the development and optimization of novel drug‐linkers. Our rationale approach starts with analyzing commercial substrates and a known inhibitor with different binding stoichiometries to identify the most favorable configuration and the most stable conformation of the enzyme. Based on the binding mechanism of HGUSB to these well‐known ligands, the interaction with commercial linkers was evaluated, providing a structural determination of the recognition mechanism between the enzyme and ADCs. These findings provide valuable insights into linker‐enzyme interactions, paving the way for the rational design of novel, more efficient ADC linkers, ultimately contributing to the development of targeted therapeutic strategies.

## 2 Materials and Methods

### 2.1 HGUSB Structure Optimization and Generation of HGUSB::Inhibitor Complexes

The crystal structure of HGUSB was obtained from the Protein Data Bank (PDB) with the PDB code 3HN3 [3]. This structure was used as the starting point for the subsequent computational studies. The Molecular Operating Environment (MOE) program version 2022.02 [22] was used to prepare the protein structure. The preparation steps are based on the elimination of cofactors, other heteroatoms, water molecules and N‐linked glycans. According to the literature, oligosaccharide chains are not necessary to sustain enzymatic activity post tetramerization [23] and, for this reason, N‐glycans were not considered in this study. Moreover, potential flaws in the structure, including missing atoms or residues were fixed and the residues' protonation state was adjusted to mimic the physiological pH environment of 5.4 by adding hydrogen atoms with the “Protonate3D” module. Energy minimization of the optimized protein structure was performed using a root mean square (RMS) gradient of 0.001 kcal/mol/Å^2^ and Amber10:EHT force field [24] for system parametrization. The 3D structure of *E. coli* beta‐glucuronidase complex with the inhibitor uronic isofagomine (UIFG) (PDB code 6LEG) [25] bound in a 1:4 stoichiometry was used to correctly position the UIFG in the HGUSB binding sites. The calculation of the sequence similarity, the alignment and superposition, between the two crystallographic structures was performed using the MOE “Align and superpose” tool. Then, the obtained complex system was processed to generate four other complexes with the inhibitor in different stoichiometries: 1:1, 1:2 and 1:4, as reported in Table S1. The 1:1 and the 1:4 complexes include one HGUSB in its tetrameric form and one or four UIFG molecules, respectively. Regarding the 1:2 configuration, different combinations were simulated placing the inhibitor in A‐B, A‐C and A‐D binding sites to evaluate the effect of different site occupancies on the enzyme behavior. All these systems were submitted to MD simulations as reported below.

### 2.2 Ligand Docking

Molecular docking simulations were carried out using the MOE 2022.02 “Dock” tool for five commercial substrates that were retrieved from G‐biosciences website [26] and for eight beta‐glucuronide linkers that were retrieved from AxisPharm website [27] and another linker from the Merck company, the maleimide‐containing hydrophilic β‐glucuronide [28], whose chemical structure is reported in Figures S1 and S2. The five substrates were downloaded from PubChem database [29], whereas the linkers were drawn using the “Molecule Builder” tool by MOE 2022.02. After that, all the compounds were processed by MOE 2022.02 via the “Wash” tool that applies a set of rules for ligand preparation, like removing extraneous salts or fixing protonation states, regenerates 3D coordinates and then performs a minimization step. The obtained washed database contains 14 structures that were submitted to the “Conformational Search” tool to evaluate the most energetically favored configurations. A pool of conformers was generated for each ligand with the Low‐Mode conformational search algorithm with the default setting. The binding site is known in the literature [17] and includes Asp207, Glu451, Tyr504 and Glu540. However, to identify it properly, the “Site finder” tool was used, and dummy atoms were generated. For the docking calculations the receptor was treated as rigid, whereas the conformational space was explored for the ligands. Triangle Matcher was applied as placement methodology with the London dG scoring function, while for the refinement step the rigid receptor method was applied generating up to 5 poses for each ligand. The resulting poses were then scored using the new Generalized‐Born Volume Integral/Weighted Surface area (GBVI/WSA dG) scoring function which estimates the free energy of binding of the ligand from a given pose. After the docking procedure 1304 poses were generated; the protein ligand interaction fingerprints (PLIF) [30] for protein‐bound substrates and linkers were performed, using MOE software [22], with the scope to filter the poses according to their interaction with binding site residues. The interactions are classified as hydrogen bonds, ionic interactions and surface contacts, according to the residues involved in catalysis. At the end, only poses that present any interactions with Glu 540, Glu 451, Tyr 504 and Asp 207 amino acids, were selected for a total of 479 poses that were further filtered according to the docking score. Finally, the top scoring substrates and linkers were chosen for further analysis, including MD simulations.

### 2.3 MD Simulations

Three MD simulations 100 ns long of HGUSB monomer in complex with the top scoring substrate and linkers were performed to validate the docking pose, as well as a simulation of the HGUSB::inhibitor monomeric complex to stabilize the interaction with the inhibitor. Three MD simulation replicas were carried out for each different combination of tetrameric HGUSB in complex with the inhibitor, respecting the alphabetical index shown in Figure 1A and as reported in Table S1. All the systems were prepared using MOE software. The “Solvate” tool was used to solvate the system with a cubic box with TIP3P water model and NaCl 0.1 M with a buffer distance of 10 Å around the protein. At the end, using the “Dynamics” tool in the simulation panel of MOE, the MD simulation setup was configured and launched with AMBER20 [31]. Amber10:EHT forcefield was used for system parametrization, the particle mesh Ewald (PME) method was used for long‐range electrostatic interactions, the non‐bonded interactions (VdW) were truncated at a cutoff distance of 10 Å. The system temperature was maintained at 300 K using the Langevin thermostat, the pressure was set as constant at 100 kPa using a Monte Carlo barostat, and prior to the production run, an equilibration phase was performed for 200 ps in an NPT ensemble and for 100 ps in an NVT ensemble. The production of MD simulations was set to 500 ns with a time step of 2 fs.

### 2.4 MD Trajectories Analysis

The root‐mean‐square deviation (RMSD) and the root‐mean‐square fluctuation (RMSF) were computed on C‐alpha atoms of HGUSB by CPPTRAJ [32]. Subsequent analyses of MD trajectories were performed on the concatenated replicas of each system excluding the first 250 ns, which were considered as equilibration steps. The RMSD matrices for the cluster analysis for cMD were generated with CPPTRAJ [33], while the clusters were obtained using a customized script based on the GROMOS algorithm [34]. Hydrogen bond (H‐bonds) analysis was carried out by MDTraj [32] with an in‐house Python script, using a cutoff distance of 0.35 nm and a cutoff angle of 120°. Dynamic cross‐correlation (DCC) maps were produced in order to find time‐correlated protein motions. DCC was derived by calculating the covariance matrices of atomic fluctuations across the minimum energy frames from MD simulations. These covariance matrices were then normalized by their respective standard deviations. This process provides a correlation matrix that reports the interdependencies between the fluctuations of all enzyme residues, highlighting their coordinated movements [35].

## 3 Results and Discussion

### 3.1 Docking of Synthetic Substrates and Linkers to HGUSB

Five synthetic substrates (Figure S1) and nine linkers (Figures S2 and S3) were docked into the HGUSB binding site generating 1304 different docking poses. In order to process the results and to filter the most relevant poses, the PLIF tool was used, selecting only the poses that make interactions with residues Asp207, Glu451, Tyr504 and Glu540 [17], known to be essential for the binding and the catalysis, resulting in a database of 479 poses. The distribution of the poses based on the PLIF analysis and on the selection of binding residues is reported in Figure S4. Accordingly, the top scoring pose of each substrate and linker was selected from the filtered database and reported in Table 1 together with the corresponding docking score.

**TABLE 1:** Top scoring poses of each commercial substrate and linker alongside the corresponding docking score.

Acronym	Linker‐substrate	S (kcal/mol)	Molecular formula
Linker1	methyl (2S,3S,4S,5R,6S)‐3,4,5‐triacetyloxy‐6‐[2‐[3‐(9H‐fluoren‐9‐ylmethoxycarbonylamino)propanoylamino]‐4‐[(4‐nitrophenoxy)carbonyloxymethyl]phenoxy]oxane‐2‐carboxylate	−11.07	C_45_H_43_N_3_O_18_
Linker2	β‐D‐triacetylglucopyranosiduronyl methyl ester‐phenol‐β‐Alanine	−10.16	C_38_H_40_N_2_O_14_
Linker3	(2S,3S,4S,5R,6S)‐6‐(2‐{2‐[3‐(2,5‐dioxo‐2,5‐dihydro‐1H‐pyrrol‐1‐yl)propanamido]acetamido}‐4‐[({[(10S)‐10‐ethyl‐18‐fluoro‐10‐hydroxy‐19‐methyl‐5,9‐dioxo‐8‐oxa‐4,15‐diazahexacyclo[14.7.1.0^2^,^1^4.04,^13^.06,^11^.0^2^°,^2^4]tetracosa‐1,6(11),12,14,16(24),17,19‐heptaen‐23‐yl]carbamoyl}oxy)methyl]phenoxy)‐3,4,5‐trihydroxyoxane‐2‐carboxylic acid	−9.76	C_47_H_45_FN_6_O_17_
Linker4	β‐tetraacetylglucopyranoside‐glycerol	−8.26	C_17_H_26_O_12_
Linker5	β‐D‐Glucopyranosiduronic acid, 2‐amino‐4‐(hydroxymethyl)phenyl, methyl ester, 2,3,4‐triacetate	−7.92	C_20_H_25_NO_11_
Linker6	β‐D‐tetraacetylgalactopyranoside‐PEG3‐azide	−7.84	C_20_H_31_N_3_O_12_
Linker7	β‐D‐tetraacetylgalactopyranoside‐PEG2‐azide	−7.56	C_18_H_27_N_3_O_11_
Linker8	4‐Formyl‐2‐nitrophenyl‐β‐D‐Glucopyranosiduronic Acid Methyl Ester 2,3,4‐Triacetate	−7.37	C_20_H_21_NO_13_
Linker9	β‐tetraacetylglucopyranoside‐glycol	−7.23	C_16_H_24_O_11_
Substrate 1	6‐[(5‐bromo‐4‐chloro‐1H‐indol‐3‐yl)oxy]‐3,4,5‐trihydroxyoxane‐2‐carboxylic acid	−7.17	C_14_H_13_BrClNO_7_
Substrate 2	8‐Hydroxyquinoline glucuronide	−6.71	C_15_H_15_NO_7_
Substrate 3	(3S,4R,6S)‐3,4,5‐trihydroxy‐6‐phenoxyoxane‐2‐carboxylic acid	−6.03	C_12_H_14_O_7_
Substrate 4	Methyl‐beta‐d‐thiogalactopyranoside	−5.96	C_7_H_14_O_5_S
Substrate 5	3,4,5‐trihydroxy‐6‐methoxyoxane‐2‐carboxylic acid	−5.70	C_7_H_12_O_7_

**FIGURE 2:**
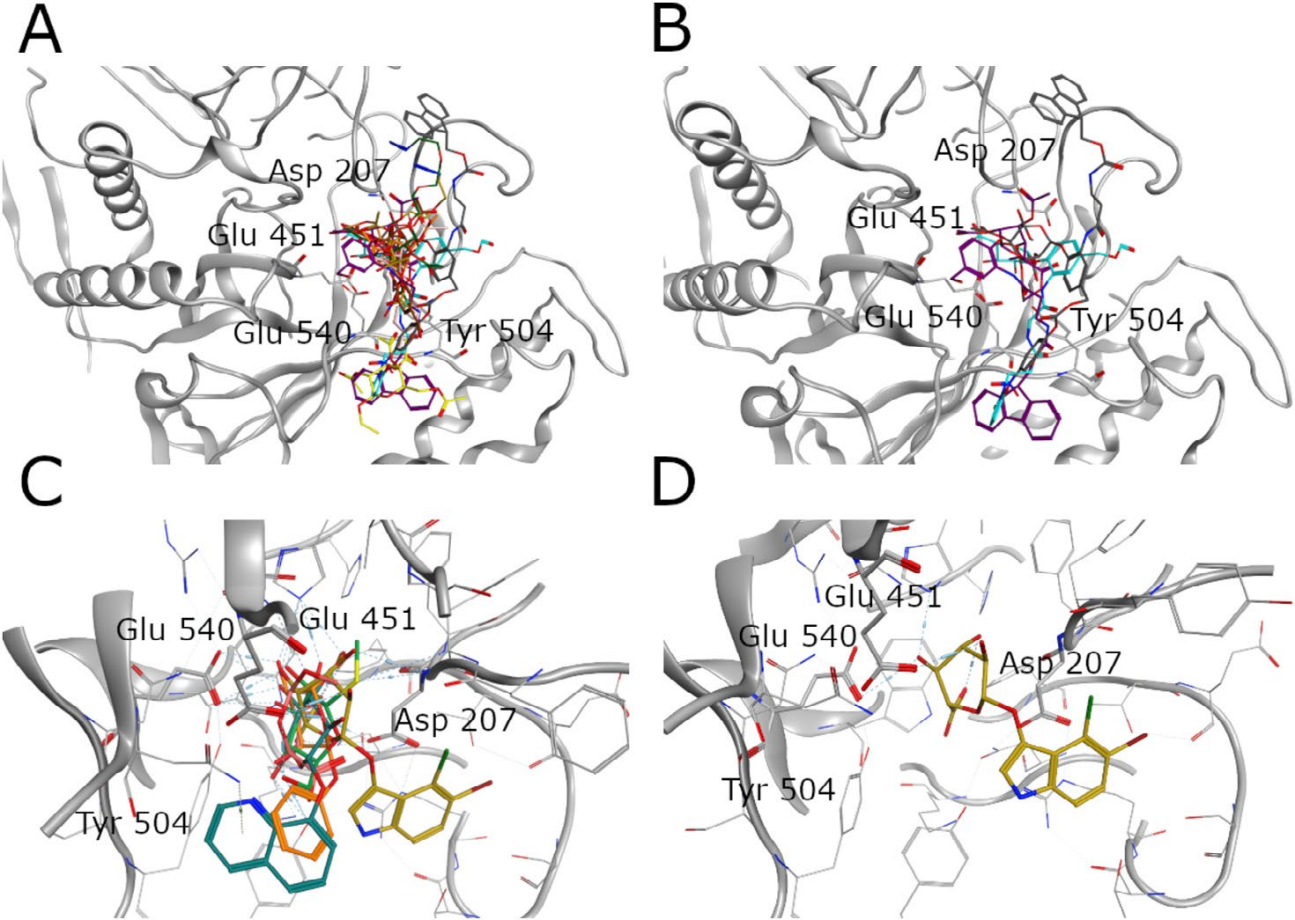
Overlay of docking results showing the binding modes of top‐scoring linkers and substrates in the HGUSB binding site. (A) The top‐scoring pose of each linker within the binding site, color‐coded for distinction. The following linkers are represented: Gray –Linker1; purple –Linker2; cyan –Linker3; orange –Linker4; pink –Linker5; green –Linker6; gold –Linker7; brown –Linker8; and yellow –Linker9. (B) Focus on the linkers with the best docking scores: Gray represents the highest‐scoring Linker 1 (docking score −11.07 kcal/mol), purple corresponds to a Linker 2 (docking score of −10.16 kcal/mol), and cyan represents a Linker 3 (docking score of approximately −9.76 kcal/mol). (C) Pose of each substrate in the binding site. The color code indicates the different commercial substrate, the substrate in gold represents Substrate 1, the one in sea green represents Substrate 2, in orange, the Substrate 3, green indicates Substrate 4, and pink represents Substrate 5. (D) 3D representation of the pose of the best docking score substrate namely Substrate 1 and shown in gold.

Docking results indicate that the linkers exhibit a higher binding affinity compared to the substrates. As shown in Figure 2A, all β‐D‐glucuronide or β‐D‐glucopyranoside units in each linker interact with key amino acid residues [17] which are essential for the hydrolysis reaction. Since the scope of this analysis was to investigate the interactions between different linker scaffolds and the binding site, the three top‐scoring linkers (docking scores of −11.07 kcal/mol, −10.16 kcal/mol, and −9.76 kcal/mol) were selected. This choice includes the two top scoring linkers with the β‐D‐glucopyranoside scaffold and the only one with the β‐D‐glucuronide scaffold ranked in third score position (Linker3). Since the other linkers also have the β‐D‐glucopyranoside unit but show lower docking score were therefore excluded from the MD study. Moreover, the top scoring substrate (docking score −7.17 kcal/mol) was selected due to its more favorable predicted binding affinities compared to the other compounds of the same class. In Figure 2B the three top‐scoring linkers were highlighted: Linker 1 in gray, Linker2 in purple, and the Linker3 (maleimide‐containing hydrophilic β‐glucuronide) in cyan. In Figure 2C the best pose for each substrate is showed in the binding site to highlight the common interaction network made by key residues [17] and the substrates, showing that the interactions with Asp207 and Tyr504 are present only in Substrate 1 and Substrate 2 binding poses. The first one (Figure 2D) resulted as the top scoring substrate.

### 3.2 Evaluation of Monomeric Complexes Stability for Substrate and Inhibitor

**FIGURE 3:**
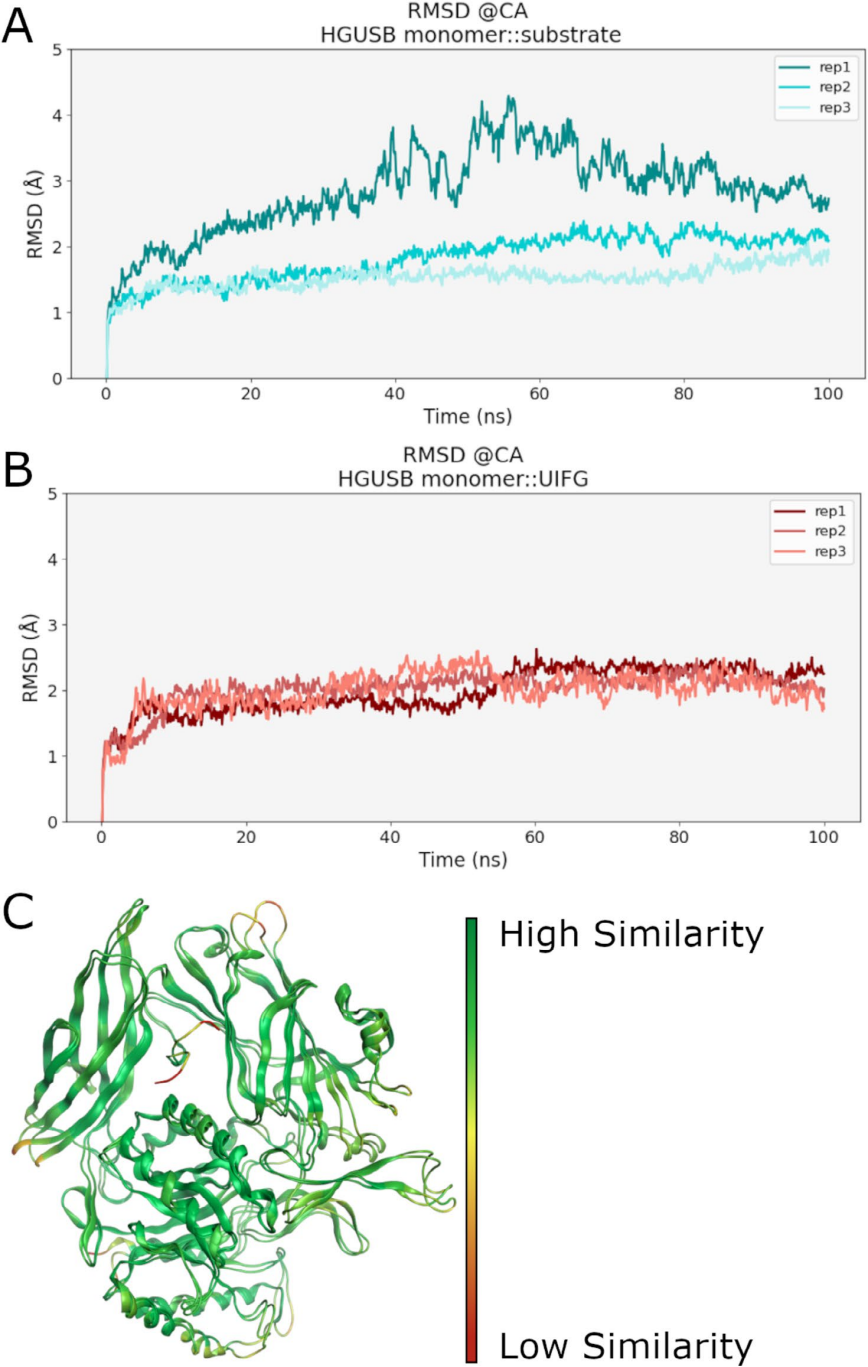
RMSD and structural analysis of HGUSB monomer::Substrate1 and HGUSB::Inhibitor systems after MD simulations. (A) RMSD plot of Cα atoms for each simulation of HGUSB monomer::Substrate1 complex. (B) and HGUSB monomer::Inhibitor complex. (C) Structural superposition of HGUSB monomer::Substrate1 and HGUSB monomer::Inhibitor complexes medoids, colored by RMSD. Regions with high deviation are shown in red and regions with low deviation (high similarity) are shown in green, while the areas with moderate deviation are indicated in yellow.

MD simulations, each lasting 100 ns, were carried out in replicates to evaluate the stability of both the monomeric HGUSB::Substrate 1 and HGUSB::UIFG complexes, where UIFG is a co‐crystallized inhibitor of the enzyme (PDB ID: 6LEG). The RMSD of the Cα atoms of the enzyme in both systems was calculated with respect to the starting frame (Figure 3A,B). For the monomer bound to the substrate, the RMSD ranged between 1.0 and 4.4 Å, while for the monomer bound to the inhibitor, it ranged between 1.0 and 2.6 Å. Notably, the substrate‐bound monomer system reached a plateau very quickly in two out of three replicas, while in replica 1 the RMSD plateau is reached only in the last 30 ns. On the other hand, the RMSD profile of the HGUSB::UIFG monomeric complex resulted very stable in all the simulations. Additionally, the most representative medoids obtained from the cluster analysis of the merged MD simulations were compared to assess structural differences. Using the MOE RMSD tool [22], after structural superposition of medoids, the structural variations were visualized by color‐coding: green indicated regions of high similarity, yellow marked areas of moderate difference, and red highlighted significant deviations. The analysis revealed no significant differences between the substrate‐bound HGUSB monomer and the inhibitor‐bound structure. Most regions are colored green, as shown in Figure 3C, indicating no structural deviation, suggesting that the substrate binding does not induce conformational changes compared to inhibitor binding.

**FIGURE 4:**
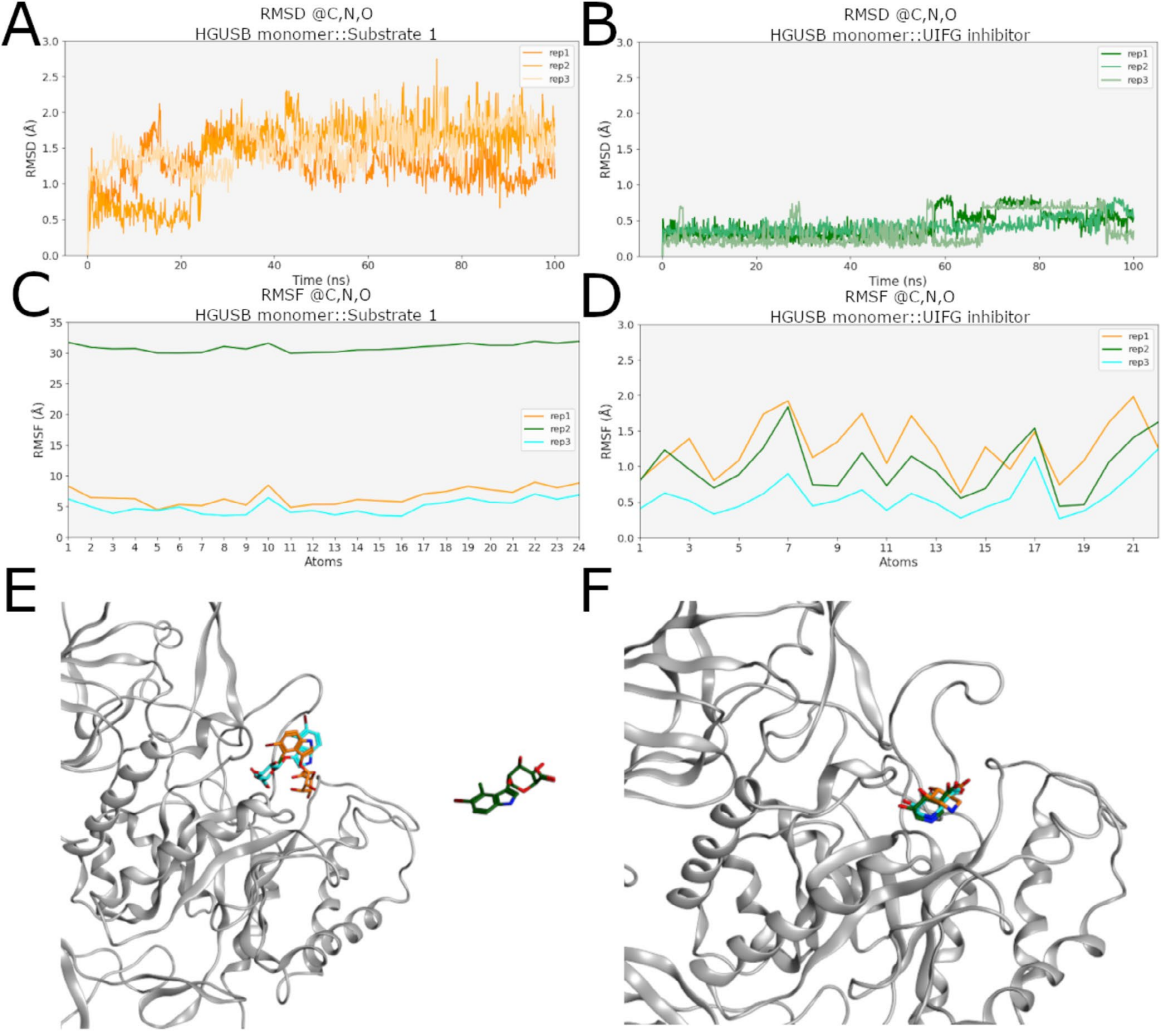
The behavior of substrate and inhibitor during MD trajectories. Cα atoms RMSD profile of (A) the substrate and (B) the inhibitor of each MD replica. RMSF plot of heavy atoms of the substrate (C), illustrating the instability of binding and its detachment from the binding site, and the inhibitor (D) demonstrating its stability in the binding pocket. Structural representation of medoids showing the position of the substrate (E) and of the inhibitor (F) with respect to the binding site. Data of the first replica are shown in yellow, data of the second replica are shown in green, and data of the third replica are shown in cyan.

This hypothesis was supported by an analysis of RMSD and RMSF profiles of the two ligands, showing that the substrate is less stable than the inhibitor. Specifically, the RMSD of the substrate is globally more variable than the inhibitor, with a big change in replica 2 from 0.5 to 1.5 Å that suggests a considerable change in its conformation (Figure 4A,B). Furthermore, the RMSF of substrate heavy atoms in this simulation oscillates around 32 Å suggesting rotation and migration of the substrate away from the docking site (Figure 4C). On the other hand, the RMSF profile of the inhibitor presents lower values in all the replicas (below 2 Å), suggesting a prolonged residence time of the molecule in the binding site (Figure 4D). This hypothesis is confirmed also by the representative structures reported in Figure 4E,F, where the substrate is located outside the binding site and the inhibitor within. According to these results, the monomeric HGUSB::UIFG complex is more stable than the monomeric HGUSB::substrate one. This behavior can be explained considering that the substrate is expected to stay inside the pocket only the time necessary to perform the enzymatic reaction, while the inhibitor should block the enzyme in an inactive form. Overall, these findings validate the use of the inhibitor as a probe for computational studies of the tetrameric HGUSB structure, enabling a robust analysis of inter‐monomers dynamic influence. For this reason, the following investigations were performed only on HGUSB::UIFG complexes and on HGUSB in its *apo* form.

### 3.3 Structural Behavior of Tetrameric HGUSB in *Apo* and *Holo* Forms

Three independent MD simulations were conducted to study the tetrameric HGUSB in its *apo* form. Each simulation was run for 500 ns, with the RMSD profiles of Cα atoms reaching a plateau at relatively low values (< 2 Å) and stabilizing after 250 ns, indicating that the system achieved structural stability (Figure S5A). The RMSF profile revealed noticeable mobility at the protein termini and in loops located between residues 141–201 and residues 231–321, while fluctuations in the remaining portions of the protein were ≤ 2 Å (Figure S5B). This specific region was previously identified in the monomer MD simulations as having structural differences across different MD replicas (Figure 3). After concatenating the last 250 ns of each simulation, a cluster analysis was performed. The cluster matrix and the medoid structure of the most populated clusters are displayed in Figure S5C,D. The results demonstrated the structural and conformational stability of the protein, with the three medoids very similar in terms of RMSD and only small differences in some loop regions.

**FIGURE 5:**
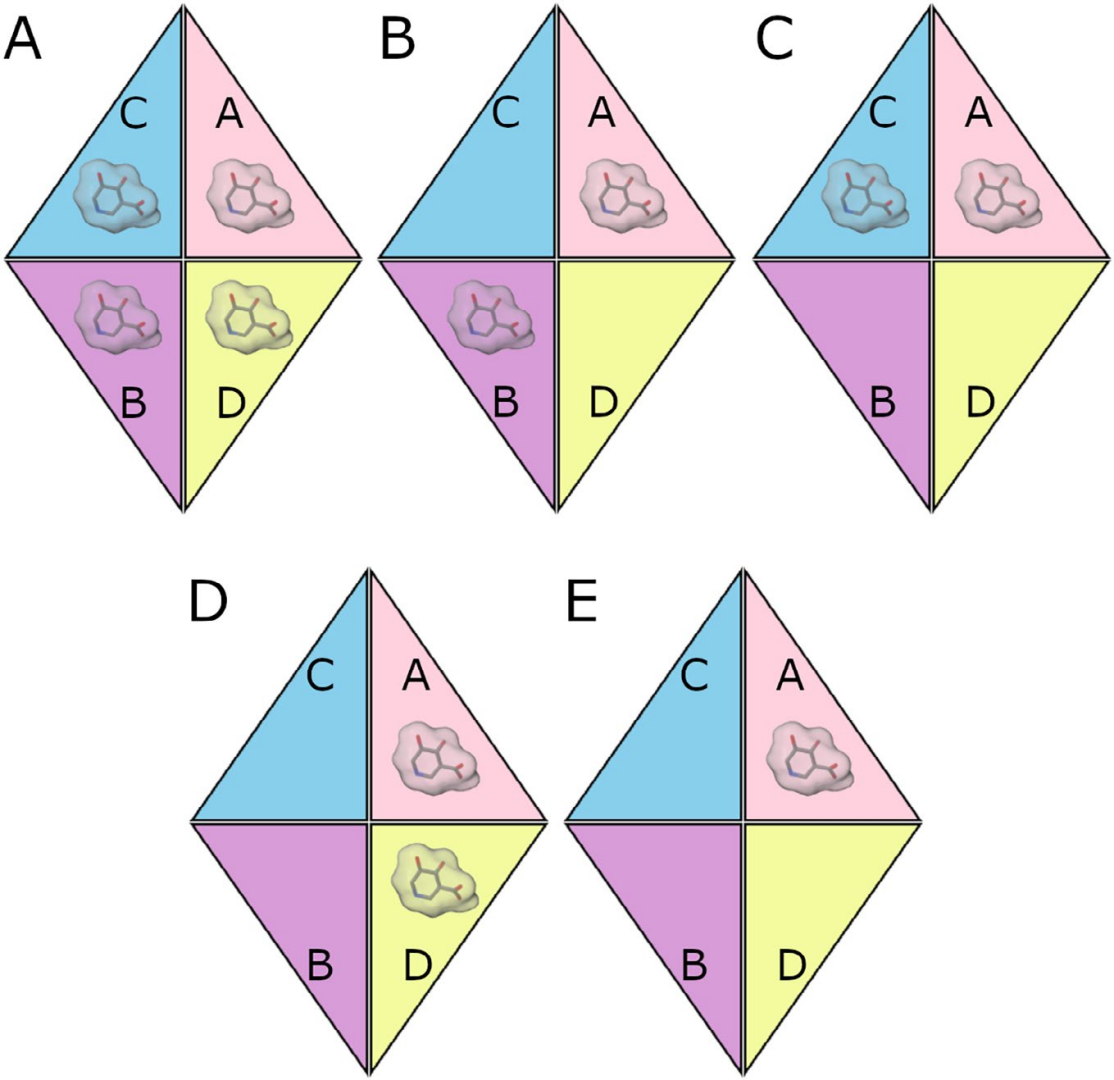
Configuration of the five different scenarios explored via MD simulations. (A) Fully occupied enzyme with the inhibitor binding to all four binding sites of the tetramer in a 1:4 stoichiometry. Half‐occupied enzyme with the inhibitor binding to monomers A and B (B), A and C (C) and A and D (D), all in a 1:2 stoichiometry. (E) Inhibitor binding to monomer A exclusively (1:1 stoichiometry).

To gain more insights into the system, 3 MD simulations were conducted in replicates considering five different configurations, with the inhibitor placed in different monomers. We started with the fully occupied enzyme configuration where the inhibitor binds to all four binding sites (HGUSB::UIFG (1:4)), followed by partially occupied configurations in which the inhibitor binds to two out of four monomers in an alternate way (HGUSB::UIFG A‐B (1:2), HGUSB::UIFG A‐C (1:2), HGUSB::UIFG A‐D (1:2)), and finally, the configuration where the inhibitor binds only to a single monomer (HGUSB::UIFG A (1:1)). All the simulated conditions are schematized in Figure 5.

**FIGURE 6:**
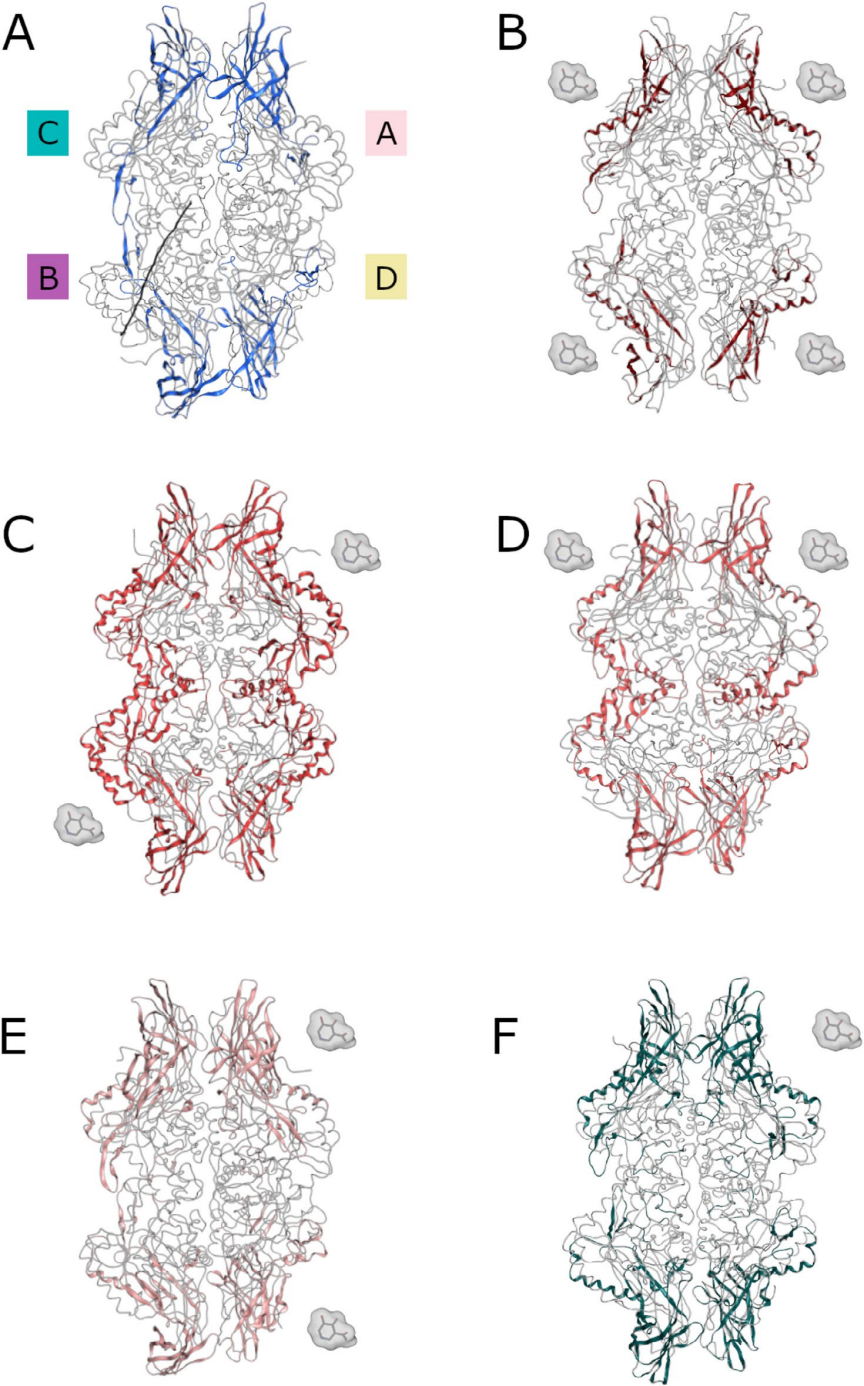
Different configurations of the HGUSB::UIFG complexes highlighting residues with high flexibility with a RMSF values of 2 Å or higher. (A) HGUSB *apo* form and its residues with high flexibility highlighted in blue. (B) HGUSB::UIFG (1:4) configuration, with residues exhibiting high flexibility shown in brown. (C) HGUSB::UIFG (1:2) A‐B configuration, showing residues with high flexibility in red. (D) HGUSB::UIFG (1:2) A‐C and residues with high flexibility in orange. (E) HGUSB::UIFG (1:2) A‐C configuration, showing amino acids with high flexibility in pink. (F) HGUSB::UIFG (1:1) A configuration, highlighting in green residues with high flexibility. In all the panels monomer A is shown in top right, monomer B is shown in bottom left, monomer C is shown in top left, and monomer D is shown in bottom right.

The behavior of all the complexes was evaluated according to geometric descriptors, that is, RMSD and RMSF as reported more in detail in Figures S6–S10. Based on this analysis, residues exhibiting higher flexibility, indicated by RMSF values of 2 Å or greater, are highlighted. In the HGUSB *apo* configuration and the HGUSB::UIFG (1:4) complex, only minor residue fluctuations were observed (Figure 6A,B), concentrated at the N‐termini of the enzyme. However, as shown in Figure 6C,D, in the 1:2 configurations (A‐B and A‐C), residue fluctuations extend deeper into the enzyme's core. In contrast, the 1:2 (A‐D) and 1:1 (A) configurations (Figure 6E,F) showed increased fluctuations around the N‐terminal regions and in the loop residues (141–201). The configuration in which the enzyme binds the inhibitor in A and B subunits (1:2 stoichiometry) resulted in the less stable one. This is inferred by both the RMSD profile of the enzyme and the RMSF profile of the inhibitor that results very flexible and unstable with peaks of more than 25 Å. In all the other configurations, namely 1:4, 1:2 subunits A‐C, 1:2 subunits A‐D, and 1:1 subunit A, instead, the RMSD profile of the enzyme is always more stable. On the other hand, the inhibitor can reach different fluctuation values based on its position, highlighting a sort of compensation effect only when it is bound to close subunits. When the binding occurs in oppositely located monomers (i.e., A‐B) this effect is lost, and one of the two ligands fluctuates more than the other. Also, in the case of the fully occupied enzyme (1:4 stoichiometry) this compensation can be observed because the fluctuation of inhibitors is balanced in pairs, for example A‐C and B‐D positions influence each other.

This analysis suggests that when ligands bind to adjacent monomers, their structural influence remains localized, minimizing global conformational changes within the tetramer. To better investigate this aspect, DCC analysis was used, as explained below.

#### 3.3.1 Analysis of DCC in the Simulated Systems

To study the effects of UIFG on different regions of the HGUSB enzyme, the differences, in terms of interdomain cross correlations, between the *apo* form and inhibitor‐bound configurations, were analyzed using DCC analysis. This technique allows the evaluation of the coordinated movements among HGUSB residues. As shown in Figure S11, the homo‐tetrameric structure of the enzyme produces a symmetric correlation graph, which can be divided into two identical mirror‐image halves, allowing us to focus on one half for a more detailed analysis.

**FIGURE 7:**
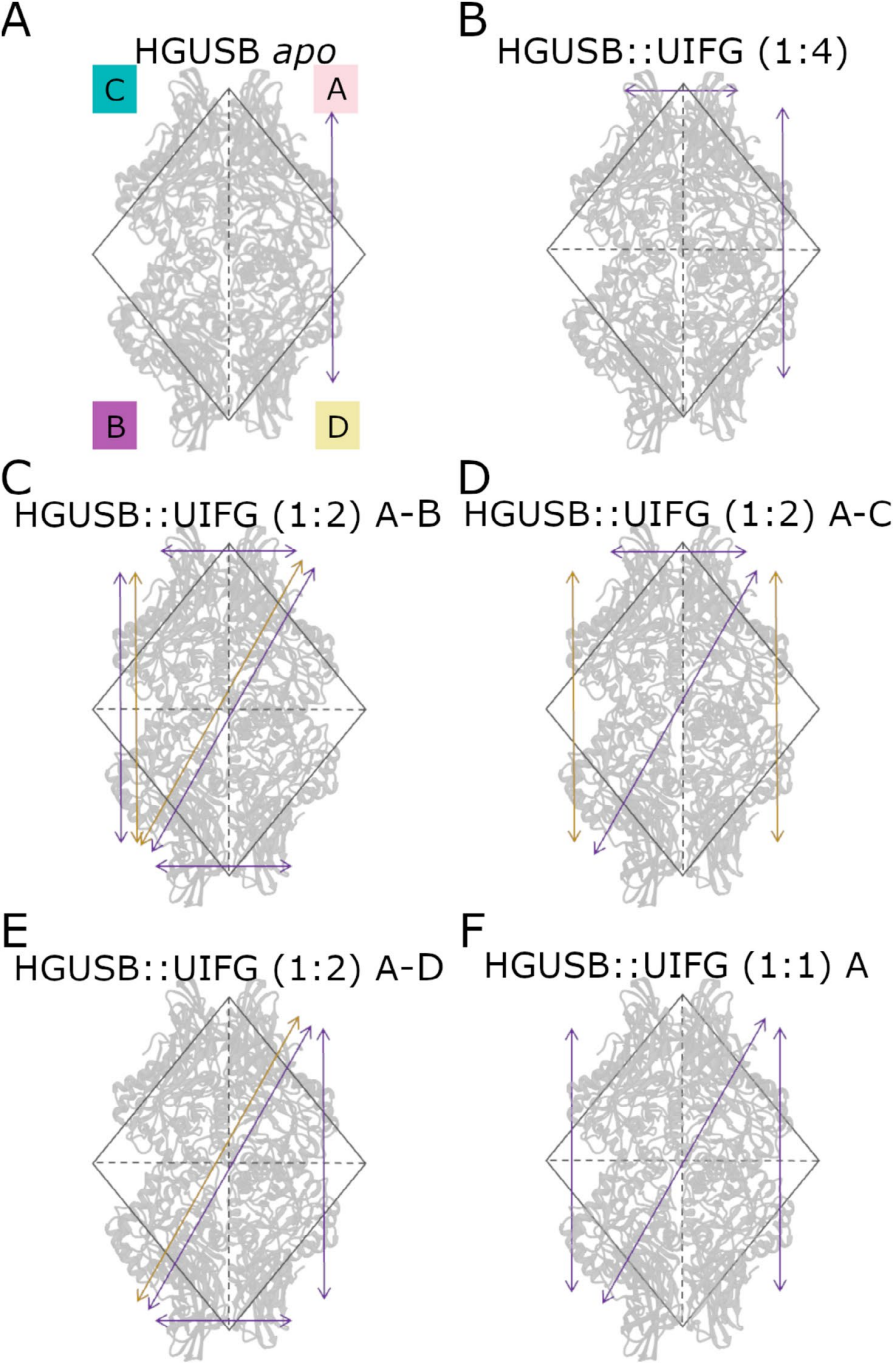
Correlation pattern of different HGUSB complexes. (A) Correlation pattern for HGUSB *Apo*. (B) Correlation pattern for HGUSB::UIFG inhibitor (1:4). (C) Correlation pattern for HGUSB::UIFG inhibitor A‐B (1:2). (D) Correlation pattern for HGUSB::UIFG inhibitor A‐C (1:2). (E) Correlation pattern for HGUSB::UIFG inhibitor A‐D (1:2). (F) Correlation pattern for HGUSB::UIFG inhibitor A (1:1). Gold and purple arrows represent positive and negative correlations, respectively. In all the panels monomer A is shown in top right, monomer B is shown in bottom left, monomer C is shown in top left, and monomer D is shown in bottom right.

DCC values range from −1 to +1, representing negative and positive correlations, respectively. Covariance analysis based on Cα atoms motions was employed to identify correlations between the movements of monomers, comparing the *apo* form with different inhibitor‐bound configurations. In the *apo* form, correlations (anti‐correlation) are observed only between monomers A–D (Figure S11A). However, in the presence of UIFG in 1:4 stoichiometry, as shown in Figure S11B, the number of residues that anticorrelate increased, indicating that the domains start influencing each other. In fact, strong anti‐correlations were observed in both the vertical (A–D) and horizontal (A–C) directions, indicating that the simultaneous presence of these movements likely helps keep the enzyme in an inactive state. The other monomers predominantly showed non‐correlated movements. In the configuration where UIFG was bound to monomers A and B (Figure S11C), an increase in both correlated and anti‐correlated movements was also noted. Particularly positive correlations were observed along the diagonal (A–B) and vertical (B–C) axes and anticorrelations were observed in diagonal (A–B), vertical (B–C), and horizontal (A–C), (B–D) axes suggesting an effect of the different binding configurations on the movements of the enzyme. For other configurations, HGUSB::UIFG A–C (1:2) and HGUSB::UIFG A–D (1:2), similar patterns of anti‐correlations were observed across the horizontal, vertical, and diagonal directions as shown in Figure S11D,E. In all these configurations the simultaneous anticorrelation between A–D or B–D and A–C or B–D is always present except for the HGUSB::UIFG A–C (1:2) indicating that in these conditions HGUSB should be inhibited. The configuration with a single inhibitor bound to monomer A (Figure S11F), however, showed anti‐correlations primarily in the vertical (B–C) and diagonal (A–B) directions, along with some non‐correlated movements, indicating that the propagation of localized movements to other subunits is not favored. These findings suggest that the inhibitor can induce distinct structural responses depending on the binding configuration but also confirm the vertical and horizontal compensation effect between close subunits (i.e., A–C and A–D), when they are occupied, as already observed comparing the fluctuations of the ligand. In Figure 7 a schematic representation of identified correlations is reported. Overall, the presence of inhibitors in all four binding sites locks the enzyme movement, stabilizing it so that each subunit works independently, leading to an inactive conformation. The *apo* form and the 1:4 configuration (Figure 7A,B), showed in fact similar correlation patterns with more pronounced vertical and horizontal correlations for the 1:4 configuration. Considering the 1:2 configurations, diagonal correlations are consistently observed as well as vertical and horizontal correlations between the bound subunits and their adjacent ones (Figure 7C–F).

**FIGURE 8:**
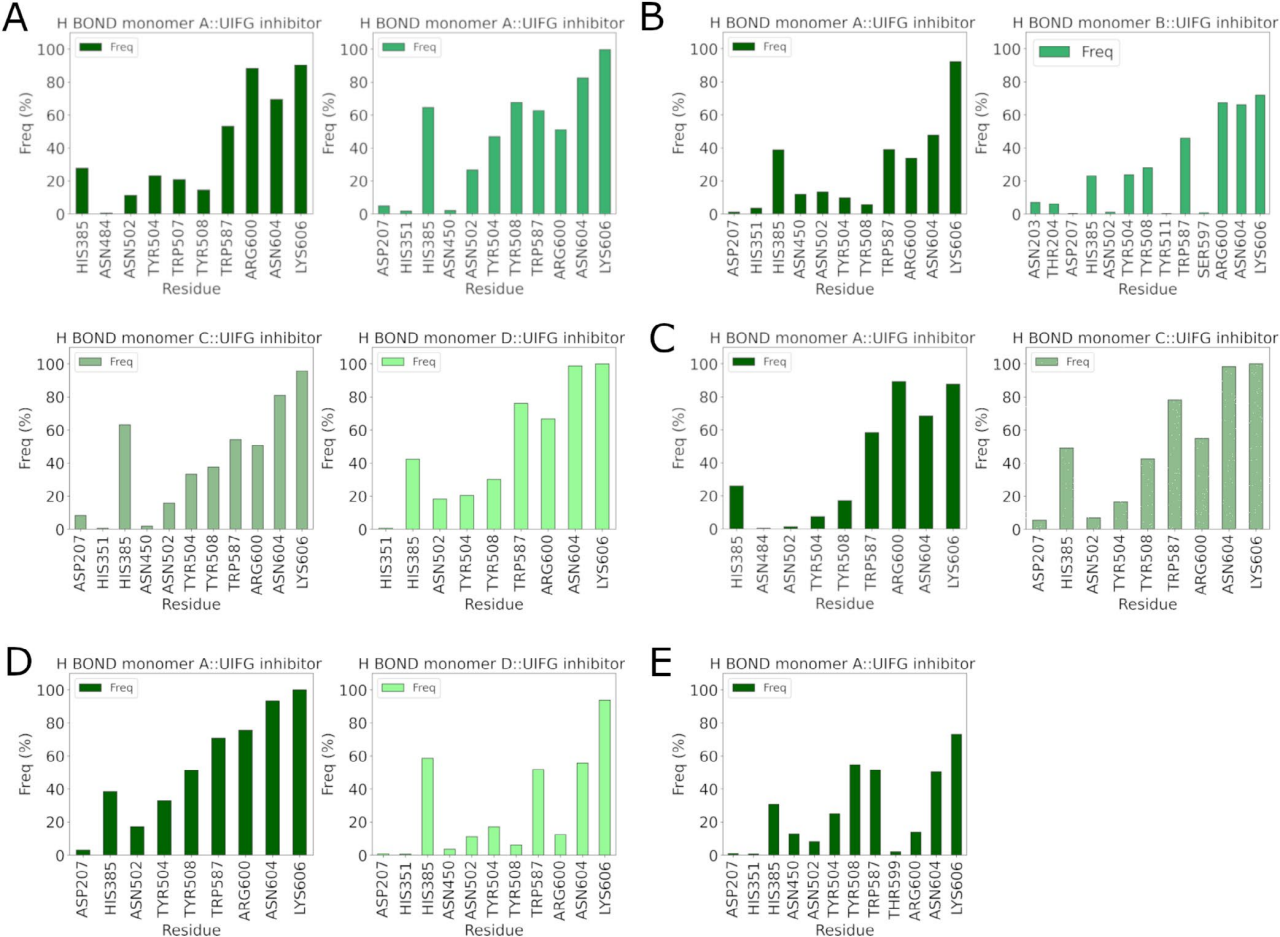
H‐bond frequencies from MD simulations of UIFG compound bound to HGUSB tetrameric complex in different stoichiometry (A) H‐bonds of HGUSB::UIFG complex in 1:4 stoichiometry; (B) H‐bonds of HGUSB::UIFG A‐B complex in stoichiometry 1:2; (C) H‐bonds of the HGUSB::UIFG A‐C complex in 1:2 stoichiometry configuration; (D) H‐bonds of the HGUSB::UIFG A‐B complex in 1:2 stoichiometry; (E) H‐bonds of HGUSB::UIFG A complex in 1:1 stoichiometry configuration.

To further investigate how the position of the inhibitor within different enzyme monomers influences the stability and conformational changes of the enzyme, an analysis of H‐bonds was conducted. As shown in Figure 8A in, the fully occupied configuration, namely HGUSB::UIFG inhibitor (1:4), H‐bonds are consistently maintained during the MD simulations across all monomers. On the other hand, in the HGUSB::UIFG A‐B (1:2) inhibitor configuration (Figure 8B), while the number of H‐bonds and the interacting residues within the enzyme remain stable, a decrease in bond frequency is noted in both monomers. In the HGUSB::UIFG A‐C (1:2) inhibitor configuration (Figure 8C), H‐bonds demonstrate consistent bonding for both monomers with the bound inhibitor throughout the simulations. Conversely, in Figure 8D (HGUSB::UIFG (1:2) A‐D inhibitor configuration), H‐bonds in monomer A remained stable throughout the MD simulations, while monomer D exhibited a decrease in H‐bond frequency over the simulations. In the HGUSB::UIFG A (1:1) inhibitor configuration (Figure 8E), although H‐bonds are preserved, there is a notable decrease in their occurrence over time during the simulations. This significant reduction in H‐bonds may impede the transmission of conformational changes within the enzyme or negatively impact the overall stability of the HGUSB structure. All these data, taken together, suggest that when ligands bind to adjacent monomers, their structural influence remains localized, minimizing global conformational changes within the tetramer. Therefore, studying a single monomer is sufficient to capture the key conformational transitions induced by the ligand, without the need to simulate the entire tetrameric complex. This approach simplifies computational analysis while preserving the accuracy of structural dynamics relevant to enzymatic function.

### 3.4 Assessment of Linker Stability Using Monomeric HGUSB Model

According to what was previously demonstrated, the stability of β‐glucuronide linkers was evaluated through MD simulations on a monomeric HGUSB system. This approach was justified by several observations. Firstly, RMSD analyses of the tetrameric complex indicated that ligand‐binding effects were primarily localized and varied depending on whether adjacent or non‐adjacent monomers were involved. While inhibitors influenced inter‐subunit interactions, β‐glucuronic linkers—due to their larger size and steric constraints—were unlikely to form stable, simultaneous interactions with multiple monomers. RMSD and RMSF analyses further confirmed that detachment events were confined to individual subunits, suggesting that inter‐monomer interactions play a minimal role in determining linker stability. Given these considerations, the monomeric model was considered an efficient and mechanistically appropriate system for analyzing linker binding in detail, while also significantly reducing computational cost. Based on these findings, all subsequent analyses involving commercial β‐glucuronidase linkers were conducted on the monomeric HGUSB structure, using the docking results as the starting point.

### 3.5 Evaluation of Monomeric Complexes Stability for the Top Three Linkers

**FIGURE 9:**
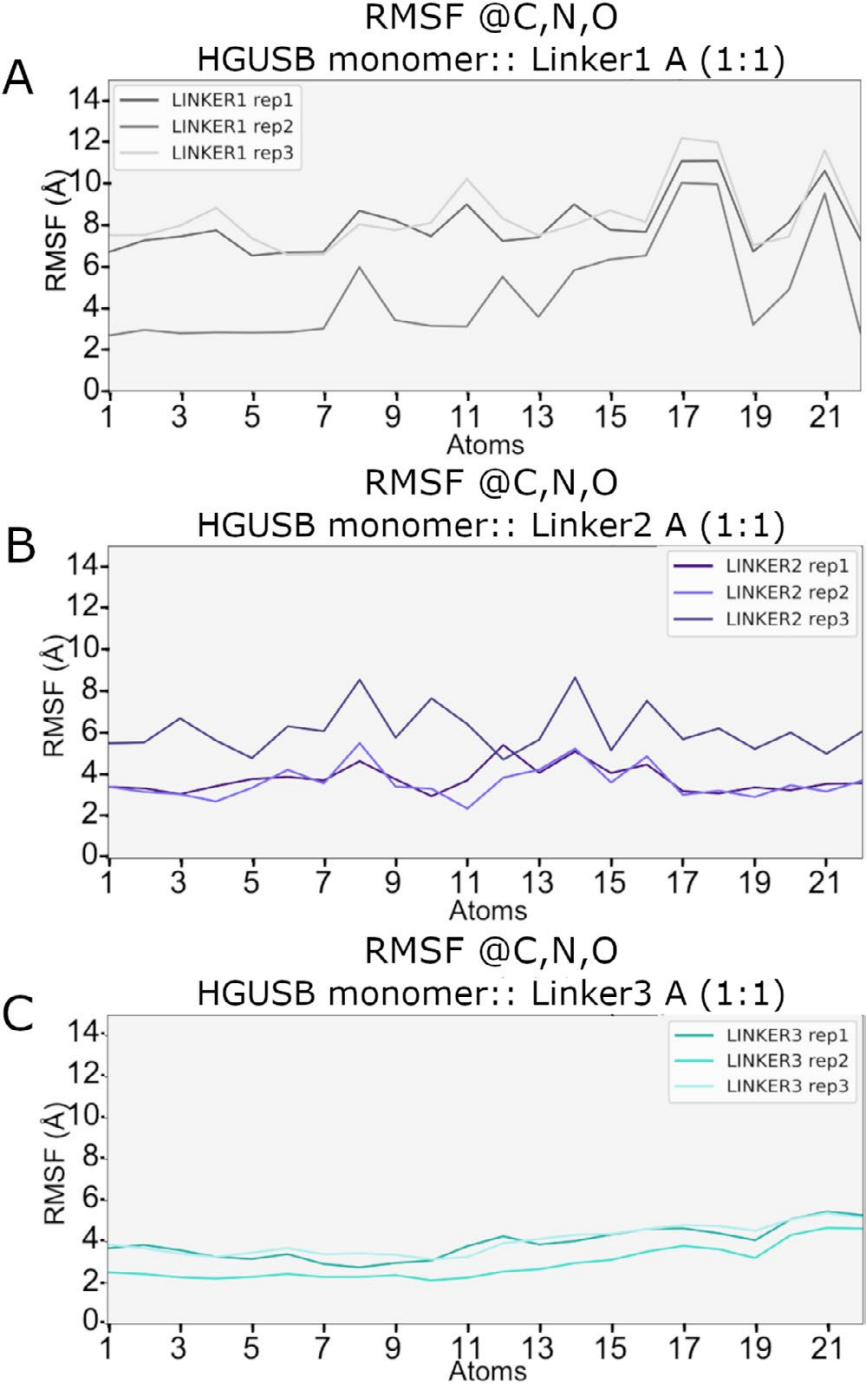
RMSF plot on heavy atoms HGUSB::Linker complexes. (A) RMSF plot for HGUSB::Linker1 (B) RMSF plot for HGUSB::Linker2 (C) RMSF plot for HGUSB::Linker3.

MD simulations, each lasting 100 ns, were carried out in three replicates to evaluate the stability of the monomeric HGUSB::Linker complexes. The RMSD of Cα atoms was computed for each system with respect to the initial structure (Figure S12A–C). For the monomer bound to the top scoring linkers (i.e., Linker1, Linker2 and Linker3), the RMSD ranged between 1.0 and 2.0 Å. Notably, all linker‐bound monomer systems reached a plateau across all three replicas, indicating stable conformations. While the RMSD analysis of the Cα atoms suggests overall stability of the monomeric HGUSB::Linker complexes, the behavior of the ligands reveals notable differences. Specifically, the RMSD analysis of the ligands reveals that Linker1 and Linker2 do not reach a plateau (Figure S13A,B), indicating differences in binding stability compared to Linker3. In contrast, Linker3 exhibits more stable binding, as evidenced by the RMSD plateau observed after 60 ns in all replicas (Figure S13C). To further investigate the stability of the HGUSB::Linker complexes, RMSF analysis was performed for Cα, revealing no substantial fluctuation differences among the complexes (Figure S14). Moreover, RMSF analysis of the three ligands indicated that Linker1 and Linker2 were less stable than Linker3. Specifically, for Linker1, complete detachment from the binding site was observed in the first replica after 60 ns and in the third replica after 18 ns, as evidenced by RMSF values reaching around 10 Å (Figure 9A). Similarly, Linker2 also exhibited detachment from the binding site, particularly in the third replica after 80 ns, indicated by higher RMSF values (Figure 9B). In contrast, Linker3 showed minor fluctuations in the RMSF profile (Figure 9C), suggesting greater stability within the binding site.

**FIGURE 10:**
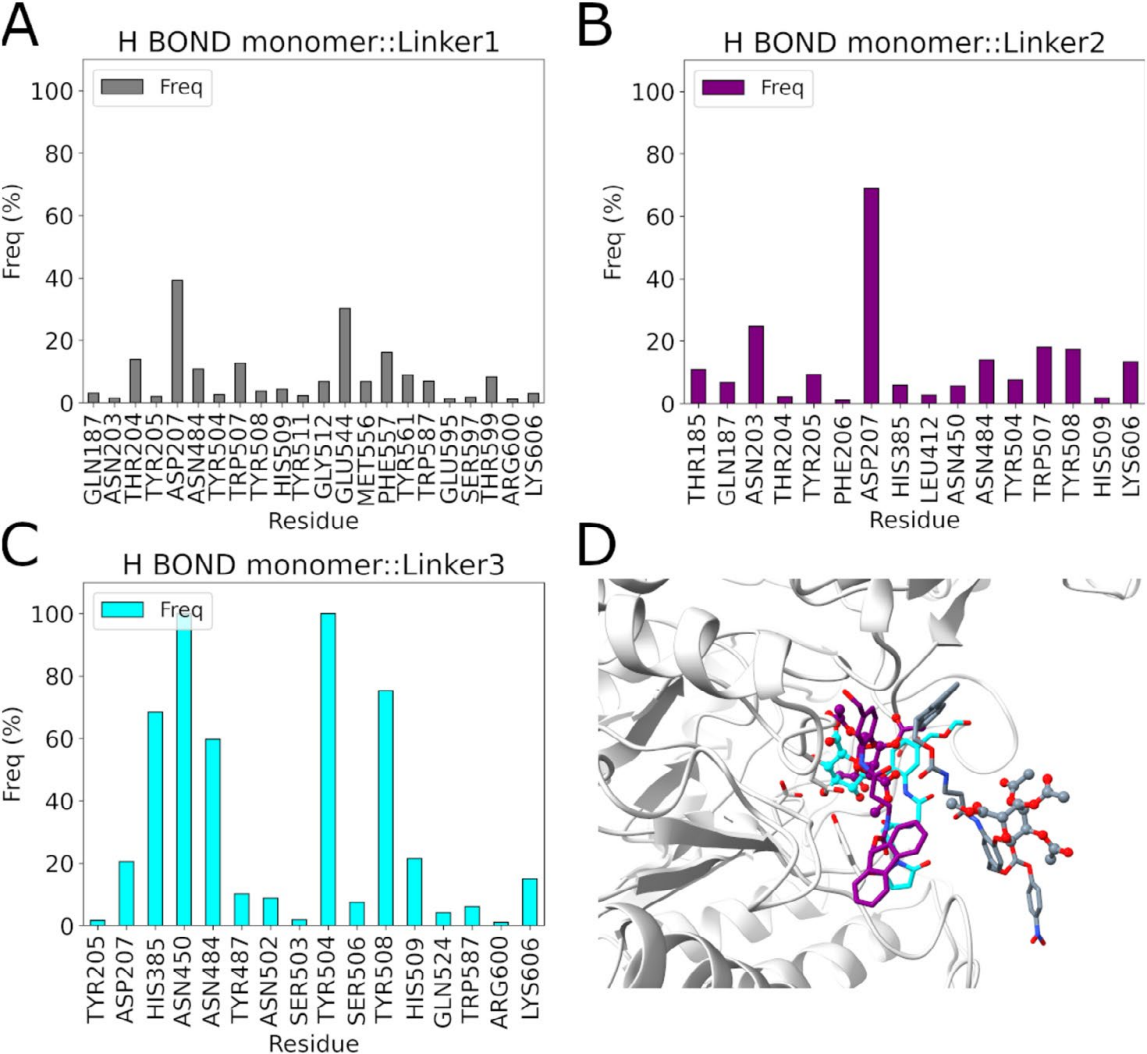
H‐bonds analysis of monomeric HGUSB::Linker systems. (A) H‐bonds HGUSB::Linker1. (B) H‐bonds HGUSB::Linker2. (C) H‐bonds HGUSB::Linker3. (D) Representative structure of Linkers, extracted from the cluster analysis, highlighting the detachment of Linker1 by the HGUSB binding site.

This increased stability of the HGUSB active site binding for Linker3, compared to the other two complexes, was further supported by the H‐bond analysis shown in Figure 10A–C. Specifically, the H‐bond analysis revealed a significant reduction in H‐bonds for Linker1 and Linker2 (Figure 10A,B), with the most frequent interactions occurring with Asp207, a key residue crucial for catalytic activity. In contrast, for Linker3, H‐bonds consistently involved Tyr504, another important residue for catalytic activity, as well as other amino acids surrounding the binding site, which further stabilized the complex. Additionally, the most representative medoids (reported in Figure S15) obtained from the cluster analysis of the merged MD simulations were compared to assess structural differences, revealing significant differences between the Linker::HGUSB complexes. Particularly, using the MOE RMSD tool [22], in Figure 10D, D‐glucuronide unit for Linker3 and the β‐D‐glucopyranoside unit for Linker1 and Linker2, respectively, are highlighted in ball and stick representation to reveal significant differences between the Linkers‐bound HGUSB monomer in the active site. As shown, only in the Linker3 (in cyan) the β‐D‐glucuronide portion binds the amino acids in the active site, while for Linker1 (in gray) a detachment from the binding site was observed and finally for Linker2 (in purple) a different orientation for the β‐D‐glucopyranoside unit was seen. These structural differences highlight the distinct binding modes and interactions of the linkers with HGUSB, which may have significant implications for their stability and activity within the HGUSB active site.

In conclusion, all these last analyses underscore the role of the β‐D‐glucuronide unit over the β‐D‐glucopyranoside one in the interaction with the active site, resulting in a more stable complex for HGUSB::Linker 3.

## 4 Conclusion

This work provides a detailed structural characterization of HGUSB in complex with both inhibitor and β‐glucuronide linkers, with implications for the design and optimization of novel ADCs. Molecular docking and MD simulations identified HGUSB::UIFG (1:4), A‐C (1:2), and A‐D (1:2) as the most stable inhibitor‐binding configurations, with A‐C (1:2) selected as a better configuration for larger ligands. To investigate linker stability, simulations were performed on a monomeric HGUSB system. This approach was driven by several factors: tetrameric simulations showed that ligand‐induced effects were mainly local and dependent on the specific monomers involved; due to the size of the linker, stable interactions between multiple monomers were sterically unlikely; and RMSD and RMSF analyses confirmed that fluctuations and detachment events were confined to single subunits. Therefore, the monomeric model provided an efficient mechanistically relevant framework for the study of alternative linkers to the active site of the enzyme. Among the candidates, linker3 showed the highest structural stability, maintaining persistent interactions with key catalytic residues, as supported by H‐bonds and clustering analyses. These results highlight the stabilizing role of the β‐D‐glucuronide unit within the HGUSB active site and provide a solid basis for the rational design of β‐glucuronide linker‐based ADCs.

## Author Contributions

**Giorgia Canini:** writing – original draft, writing – review and editing, investigation, formal analysis, data curation, conceptualization, methodology. **Simona Saporiti:** investigation, writing – original draft, writing – review and editing, formal analysis, data curation, conceptualization, methodology. **Crescenzo Coppa:** visualization, writing – review and editing, data curation. **Mara Rossi:** visualization, supervision. **Fabio Centola:** conceptualization, project administration, supervision, data curation. **Alessandro Arcovito:** conceptualization, project administration, funding acquisition, supervision, data curation.

## Supporting information


**Data S1:** prot70077‐sup‐0001‐supinfo.docx.

## Data Availability

The data that support the findings of this study are available from the corresponding author upon reasonable request.

## References

[1] F. Zhang , Z. Zhang , and R. J. Linhardt , “Glycosaminoglycans,” in Handbook of Glycomics (Elsevier, 2010), 59–80.

[2] C. W. Frevert and T. N. Wight , “Extracellular Matrix | Matrix Proteoglycans,” in Encyclopedia of Respiratory Medicine (Elsevier, 2006), 184–188.

[3] M. I. Hassan , A. Waheed , J. H. Grubb , H. E. Klei , S. Korolev , and W. S. Sly , “High Resolution Crystal Structure of Human b‐Glucuronidase Reveals Structural Basis of Lysosome Targeting,” PLoS One 8 (2013): e79687, 10.1371/journal.pone.0079687. 24260279 PMC3834196

[4] E. M. Kamel , F. F. A. Alkhayl , H. A. Alqhtani , M. Bin‐Jumah , H. A. Rudayni , and A. M. Lamsabhi , “Dissecting Molecular Mechanisms Underlying the Inhibition of β‐Glucuronidase by Alkaloids From *Hibiscus trionum* : Integrating In Vitro and In Silico Perspectives,” Computers in Biology and Medicine 180 (2024): 108969, 10.1016/j.compbiomed.2024.108969. 39089106

[5] P. Awolade , N. Cele , N. Kerru , L. Gummidi , E. Oluwakemi , and P. Singh , “Therapeutic Significance of β‐Glucuronidase Activity and Its Inhibitors: A Review,” European Journal of Medicinal Chemistry 187 (2020): 111921, 10.1016/J.EJMECH.2019.111921. 31835168 PMC7111419

[6] S. R. Bonam , F. Wang , and S. Muller , “Lysosomes as a Therapeutic Target,” Nature Reviews. Drug Discovery 18 (2019): 923–948, 10.1038/s41573-019-0036-1. 31477883 PMC7097195

[7] A. J. Anlyan , J. Gamble , and H. A. Hoster , “Beta‐Glucuronidase Activity of the White Blood Cells in Human Leukemias and Hodgkin's Disease,” Cancer 3 (1950): 116–123, 10.1002/1097-0142(1950)3:1<116::AID-CNCR2820030114>3.0.CO;2-G. 15410322

[8] E. Boyland , D. M. Wallace , and D. C. Williams , “Enzyme Activity in Relation to Cancer,” British Journal of Cancer 11 (1957): 578–589, 10.1038/bjc.1957.71. 13510516 PMC2073720

[9] S. Chuprakov , A. O. Ogunkoya , R. M. Barfield , et al., “Tandem‐Cleavage Linkers Improve the In Vivo Stability and Tolerability of Antibody–Drug Conjugates,” Bioconjugate Chemistry 32 (2021): 746–754, 10.1021/acs.bioconjchem.1c00029. 33689309

[10] W. H. Fishman and A. J. Anlyan , “Comparison of the β‐Glucuronidase Activity of Normal, Tumor, and Lymph Node Tissues of Surgical Patients,” Science 106 (1947): 66–67, 10.1126/science.106.2742.66. 17820734

[11] Y. V. Kovtun and V. S. Goldmacher , “Cell Killing by Antibody‐Drug Conjugates,” Cancer Letters 255 (2007): 232–240, 10.1016/j.canlet.2007.04.010. 17553616

[12] M. Graaf , E. Boven , H. Scheeren , H. Haisma , and H. Pinedo , “Beta‐Glucuronidase‐Mediated Drug Release,” Current Pharmaceutical Design 8 (2002): 1391–1403, 10.2174/1381612023394485. 12052215

[13] I. Tranoy‐Opalinski , T. Legigan , R. Barat , et al., “β‐Glucuronidase‐Responsive Prodrugs for Selective Cancer Chemotherapy: An Update,” European Journal of Medicinal Chemistry 74 (2014): 302–313, 10.1016/j.ejmech.2013.12.045. 24480360

[14] Y. Yang , H. Aloysius , D. Inoyama , Y. Chen , and L. Q. Hu , “Enzyme‐Mediated Hydrolytic Activation of Prodrugs,” Acta Pharmaceutica Sinica B 1 (2011): 143–159, 10.1016/J.APSB.2011.08.001.

[15] A. W. Wong , S. He , and S. G. Withers , “Synthesis of 5‐Fluoro‐β‐D−Glucopyranosyluronic Acid Fluoride and Its Evaluation as a Mechanistic Probe of *Escherichia coli* β‐Glucuronidase,” Canadian Journal of Chemistry 79 (2001): 510–518, 10.1139/v00-155.

[16] S. Jain , W. B. Drendel , Z. W. Chen , F. S. Mathews , W. S. Sly , and J. H. Grubb , “Structure of Human Beta‐Glucuronidase Reveals Candidate Lysosomal Targeting and Active‐Site Motifs,” Nature Structural Biology 3 (1996): 375–381, 10.1038/NSB0496-375. 8599764

[17] A. W. Wong , S. He , J. H. Grubb , W. S. Sly , and S. G. Withers , “Identification of Glu‐540 as the Catalytic Nucleophile of Human β‐Glucuronidase Using Electrospray Mass Spectrometry,” Journal of Biological Chemistry 273 (1998): 34057–34062, 10.1074/JBC.273.51.34057. 9852062

[18] M. R. Islam , S. Tomatsu , G. N. Shah , et al., “Active Site Residues of Human β‐Glucuronidase: Evidence for Glu540 as the Nucleophile and Glu451 as the Acid‐Base Residue,” Journal of Biological Chemistry 274 (1999): 23451–23455, 10.1074/JBC.274.33.23451. 10438523

[19] A. Sun and R. Wang , “Mucopolysaccharidosis Type VII,” (2024).

[20] C. M. Simonaro , M. D'Angelo , X. He , et al., “Mechanism of Glycosaminoglycan‐Mediated Bone and Joint Disease : Implications for the Mucopolysaccharidoses and Other Connective Tissue Diseases,” American Journal of Pathology 172 (2008): 112–122, 10.2353/AJPATH.2008.070564. 18079441 PMC2189614

[21] H. Naz , A. Islam , A. Waheed , W. S. Sly , F. Ahmad , and I. Hassan , “Human β‐Glucuronidase: Structure, Function, and Application in Enzyme Replacement Therapy,” Rejuvenation Research 16 (2013): 352–363, 10.1089/REJ.2013.1407. 23777470

[22] Molecular Operating Environment (MOE) , “2022.02 Chemical Computing Group ULC,” (2024), 910–1010 Sherbrooke St. W., Montreal, QC H3A 2R7, Canada.

[23] J. M. Shipley , J. H. Grubb , and W. S. Sly , “The Role of Glycosylation and Phosphorylation in the Expression of Active Human Beta‐Glucuronidase,” Journal of Biological Chemistry 268 (1993): 12193–12198, 10.1016/S0021-9258(19)50325-8. 8505339

[24] D. A. Case , T. A. Darden , T. E. Cheatham, III , et al., AMBER 10, ed. K. M. Merz (University of California, 2008).

[25] H. Y. Lin , C. Y. Chen , T. C. Lin , et al., “Entropy‐Driven Binding of Gut Bacterial β‐Glucuronidase Inhibitors Ameliorates Irinotecan‐Induced Toxicity,” Communications Biology 4 (2021): 280, 10.1038/S42003-021-01815-W. 33664385 PMC7933434

[26] G‐Biosciences , “G‐Biosciences,” (2023), 2023 Geno Technology Inc., USA, http://www.gbiosciences.com.

[27] CD Bioparticles , “β‐Glucuronide Linkers,” accessed 5 Mar 2025, https://www.cd‐bioparticles.net/glucuronide‐linkers?gad_source=1&gclid=CjwKCAiAiaC‐BhBEEiwAjY99qJq6RFJcxJLyBUp72lsp4D_7OMLQET7tYFUkHvLcOK04pOtp8jNhkhoCXFUQAvD_BwE.

[28] W. N. Sloot , E. Bertotti , M. Onidi , et al., “The Nonclinical Safety Assessment of a Novel Anti‐CEACAM5 Antibody Exatecan Conjugate Predicts a Low Risk for Interstitial Lung Disease (ILD) in Patients‐The Putative Mechanism Behind ILD,” International Journal of Toxicology 44 (2025): 10915818241306040, 10.1177/10915818241306039. 39754485

[29] S. Kim , J. Chen , T. Cheng , et al., “PubChem 2023 Update,” Nucleic Acids Research 51 (2023): D1373–D1380, 10.1093/nar/gkac956. 36305812 PMC9825602

[30] M. Radifar , N. Yuniarti , and E. P. Istyastono , “PyPLIF: Python‐Based Protein‐Ligand Interaction Fingerprinting,” Bioinformation 9 (2013): 325–328, 10.6026/97320630009325. 23559752 PMC3607193

[31] D. A. Case , K. A. A. Belfon , I. Ben‐Shalom , et al., “Amber 2020,” (2020), University of California, San Francisco.

[32] R. T. McGibbon , K. A. Beauchamp , M. P. Harrigan , et al., “MDTraj: A Modern Open Library for the Analysis of Molecular Dynamics Trajectories,” Biophysical Journal 109 (2015): 1528, 10.1016/J.BPJ.2015.08.015. 26488642 PMC4623899

[33] D. R. Roe and T. E. Cheatham , “PTRAJ and CPPTRAJ: Software for Processing and Analysis of Molecular Dynamics Trajectory Data,” Journal of Chemical Theory and Computation 9 (2013): 3084–3095, 10.1021/ct400341p. 26583988

[34] X. Daura , K. Gademann , B. Jaun , D. Seebach , W. F. van Gunsteren , and A. E. Mark , “Peptide Folding: When Simulation Meets Experiment,” Angewandte Chemie International Edition 38 (1999): 236–240, 10.1002/(SICI)1521-3773(19990115)38:1/2<236::AID-ANIE236>3.0.CO;2-M.

[35] S. Saporiti , D. Bianchi , O. Ben Mariem , et al., “In Silico Evaluation of the Role of Fab Glycosylation in Cetuximab Antibody Dynamics,” Frontiers in Immunology 15 (2024): 1429600, 10.3389/fimmu.2024.1429600. 39185413 PMC11342397

