# Supplementary material for: In Silico Structural Analysis of Human β‐Glucuronidase for Antibody–Drug Conjugates Optimization
Source: Proteins. 2025 Oct 31;94(3):838–52. doi: 10.1002/prot.70077 (PMC12865248; doi:10.1002/prot.70077)
Supplement: Supplementary file 1 — Data S1: prot70077‐sup‐0001‐supinfo.docx. [file PROT-94-838-s001.docx]

# Supplementary material

# In Silico Structural Analysis of Human β-glucuronidase for Antibody–Drug Conjugates optimization.

Giorgia Canini^1,†^, Simona Saporiti^2,†^, Crescenzo Coppa^1^, Mara Rossi^2^, Fabio Centola^2^ and Alessandro Arcovito^1,3,*^

^1^Fondazione Policlinico Universitario "A. Gemelli", IRCCS, Largo Agostino Gemelli 8, 00168 Roma, Italy.

^2^Analytical Excellence and Program Management, Merck Serono S.p.A., Rome, Italy.

^3^Dipartimento di Scienze Biotecnologiche di Base, Cliniche Intensivologiche e Perioperatorie, Università Cattolica del Sacro Cuore, Largo Francesco Vito 1, 00168 Roma, Italy.

†These authors contributed equally to this work

*Corresponding author: Alessandro Arcovito, mail: alessandro.arcovito@unicatt.it

**Table S1.** **The configuration of simulated systems including HGUSB tetramer in apo and holo forms.**

| **System** | **Stoichiometry** | **Binding site** | **Simulation time** | | **#Replicas** |
| --- | --- | --- | --- | --- | --- |
| HGUSB *apo* | N.A. | N.A. | 500 ns | 3 | |
| HGUSB::inhibitor | 1:1 | A | 500 ns | 3 | |
|  | 1:2 | A-B | 500 ns | 3 | |
|  | 1:2 | A-C | 500 ns | 3 | |
|  | 1:2 | A-D | 500 ns | 3 | |
|  | 1:4 | All | 500 ns | 3 | |

**
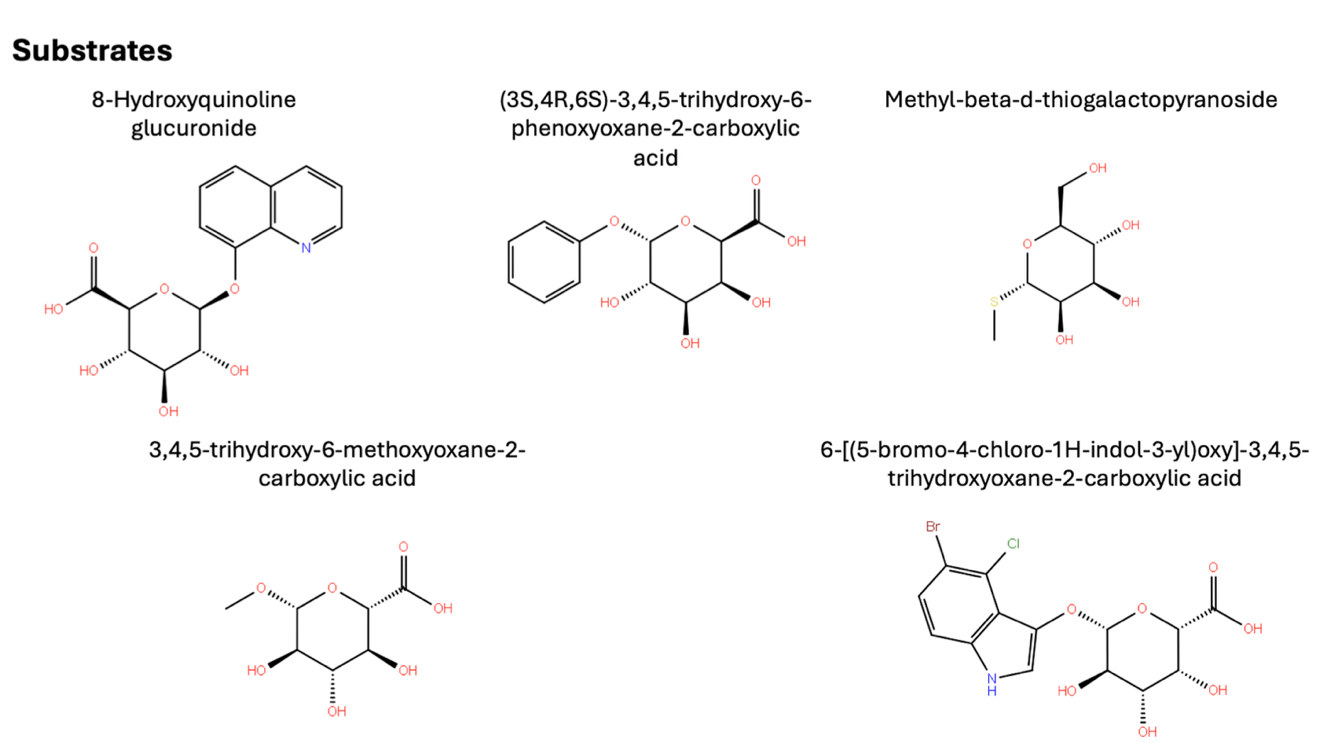
**

**Fig S1.** **Structures of the synthetic substrates used to do the docking procedure**


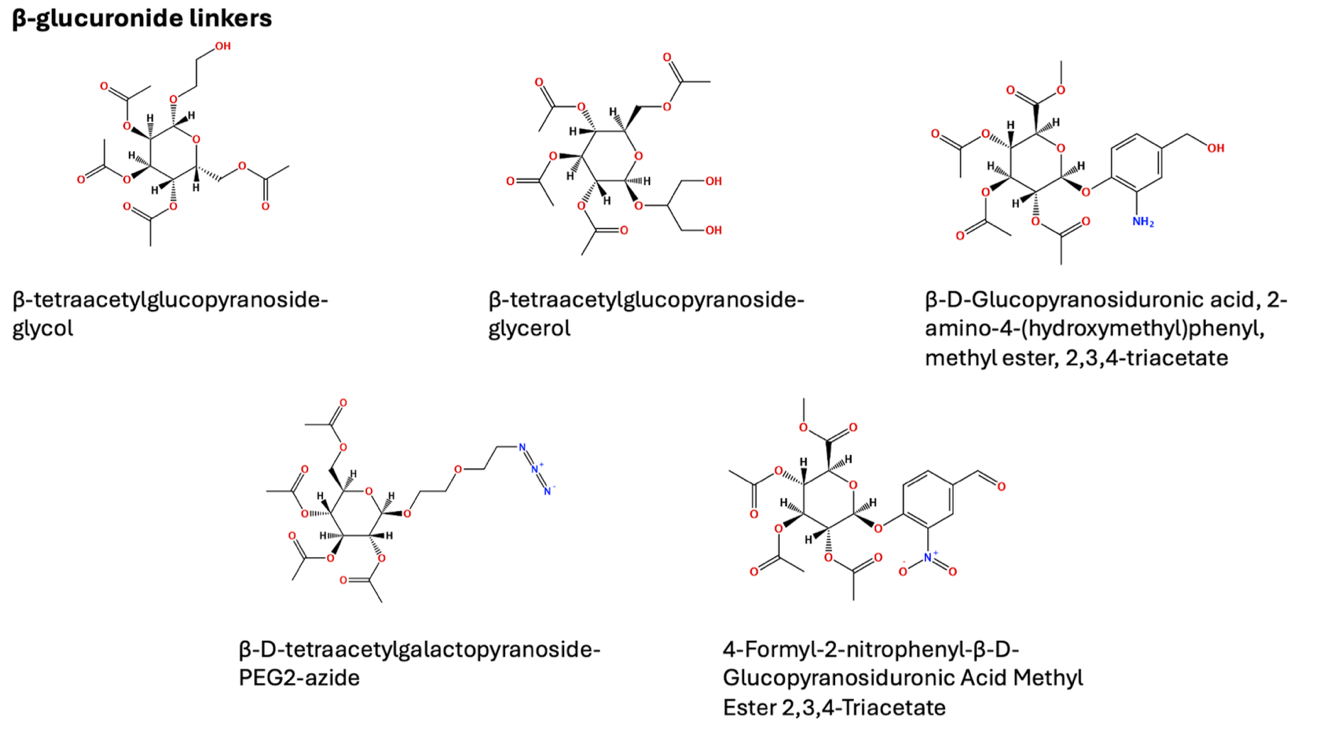


**Fig S2. Structures of the linkers from AxisPharm website used to do the docking procedure**


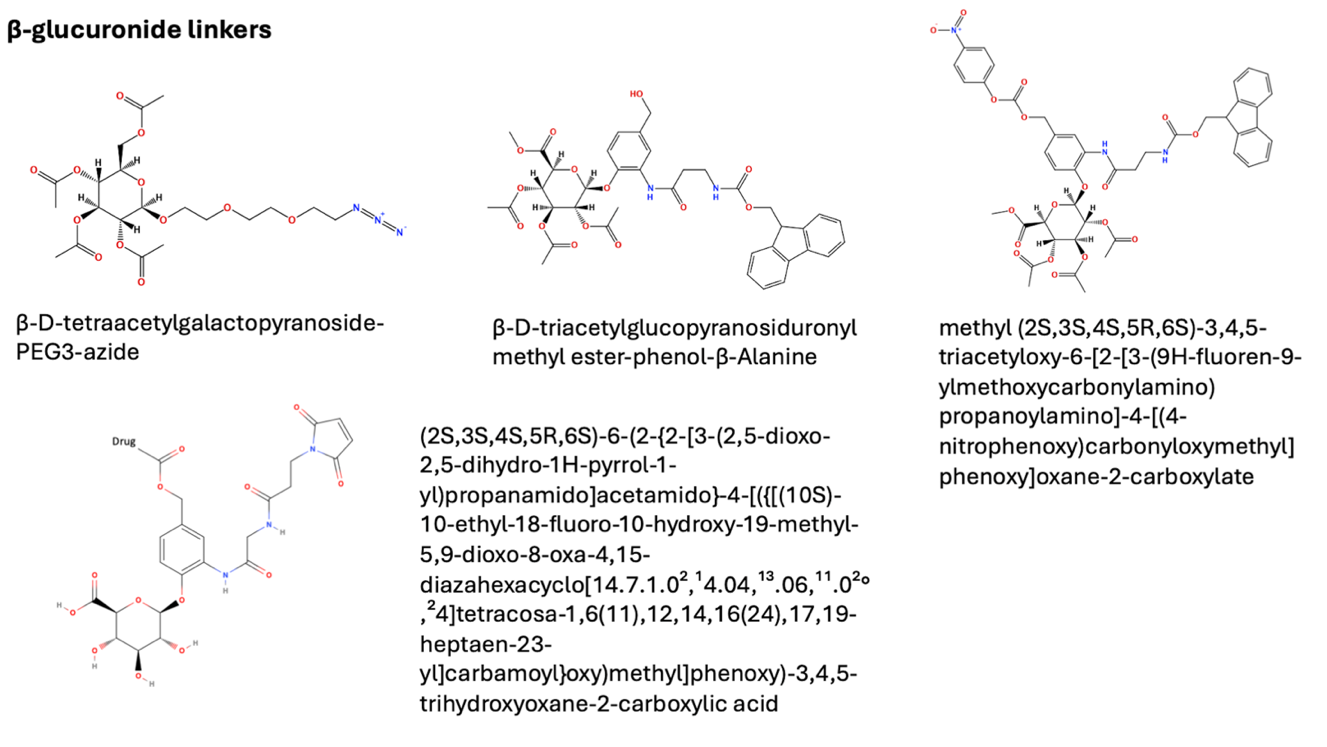


**Fig S3.** **Structures of the linkers from AxisPharm website and Maleimide-containing hydrophilic β-glucuronide linker used to do the docking procedure**


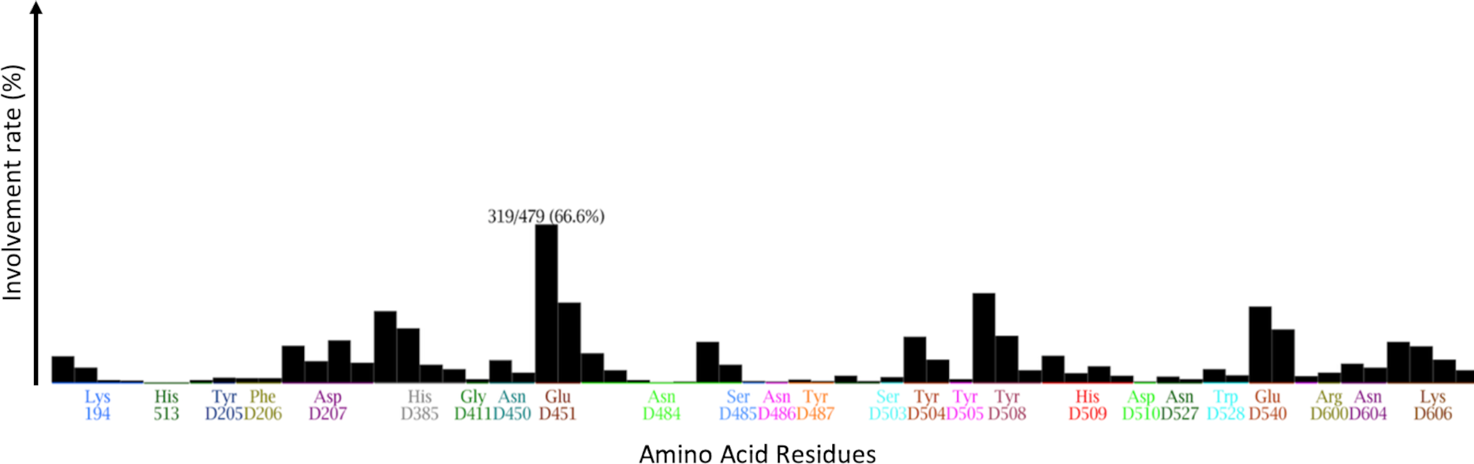


**Fig S4. Histogram reporting PLIF results showing in the Y-axis, the number of ligands, and within the residues with which they interact in the X-axis.**


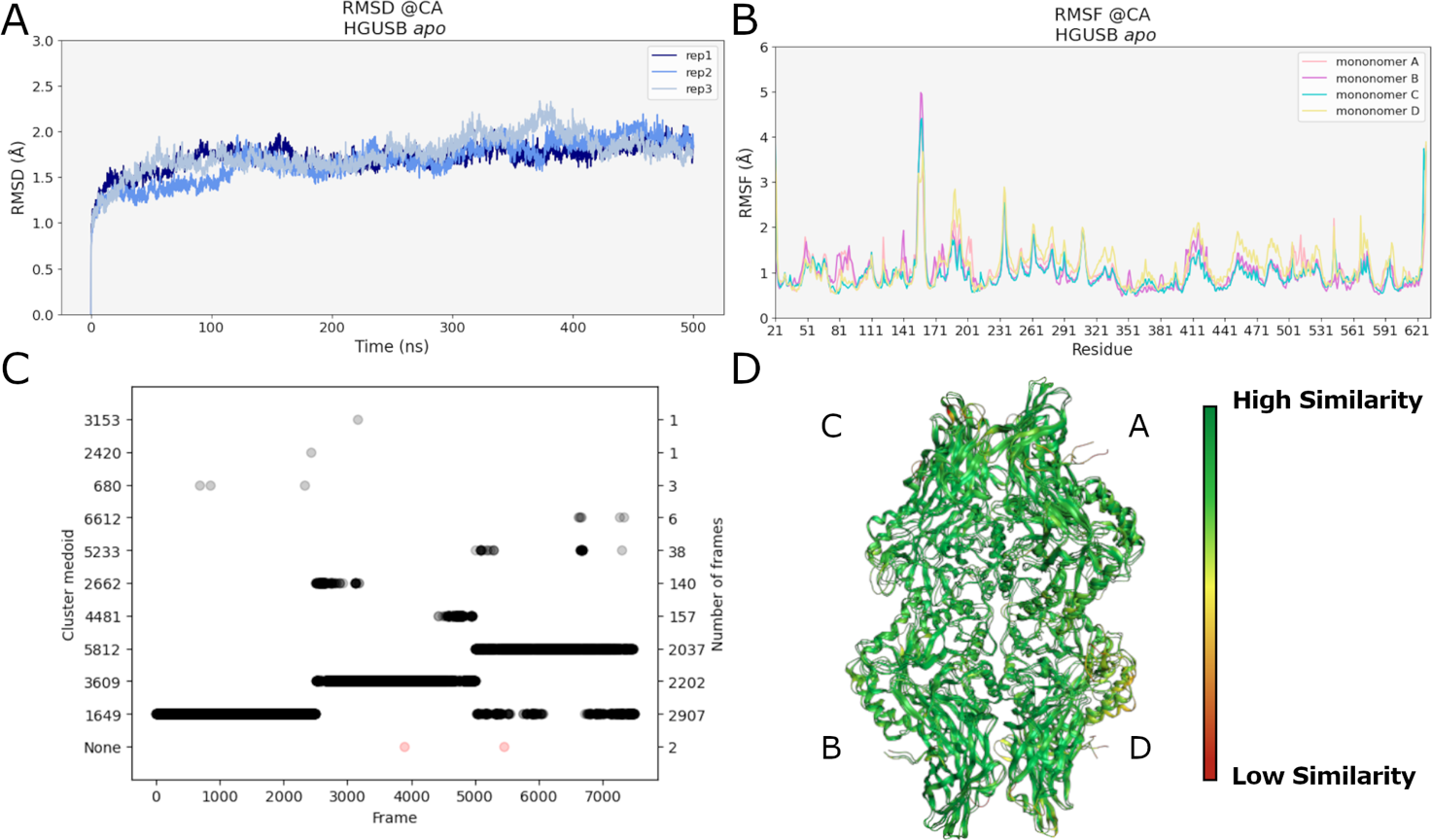


**Fig S5**. **Structural analysis of the HGUSB apo system.** (A) RMSD profile for each HGUSB apo simulation, showing that in all cases it reaches a plateau at low values (<2 Å). (B) RMSF profile for the N- and C-termini, with all residues demonstrating RMSF values ≤ 5 Å. (C) Cluster medoid of the most populated cluster. (D) Structural superposition of *apo* medoids, colored by RMSD. Regions of high deviation are shown in red and regions with low deviation and high similarity are shown in green, while the areas with moderate deviation are indicated in yellow.


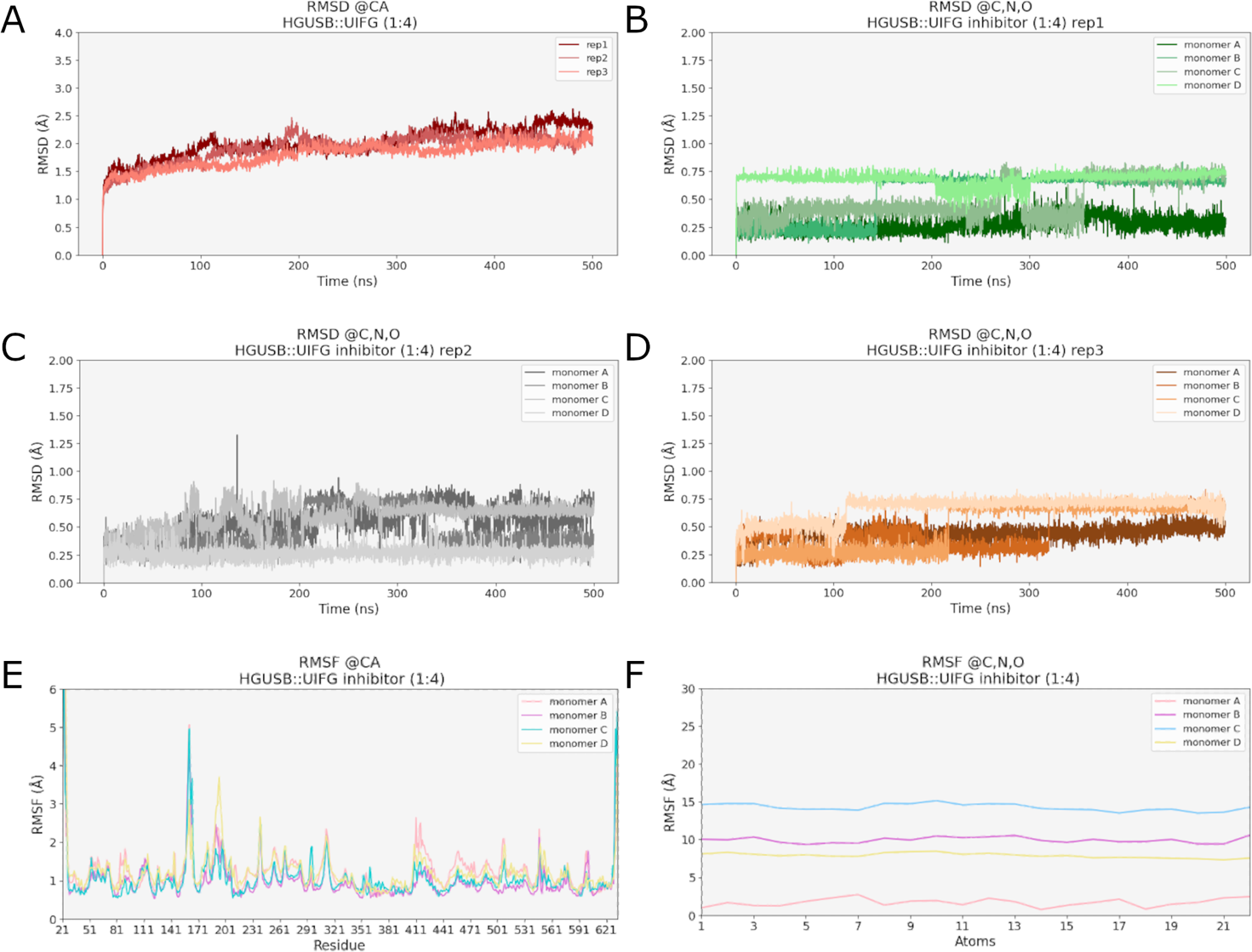


**Fig S6. RMSD and RMSF Plots of HGUSB::UIFG (1:4).** (A) RMSD plot of the Cα atoms reaching a plateau around 2.5 Å. (B) RMSD profile of the inhibitor in the first replica, showing values below 1 Å throughout the entire MD simulation. (C) RMSD profile of the inhibitor in the second replica, showing values around 1 Å during the MD simulation. (D) RMSD profile of the inhibitor in the third, showing values below 1 Å throughout the MD simulation. (E) RMSF profile of the protein from the N- and C-termini, with all residues demonstrating RMSF values ≤ 5 Å and fluctuations in similar regions as the apo-protein but with slightly lower peaks. (F) RMSF plot of the inhibitor, comparing different fluctuations when inhibitors are into all binding sites. The analysis revealed significant fluctuations for the inhibitor bound to the monomer C


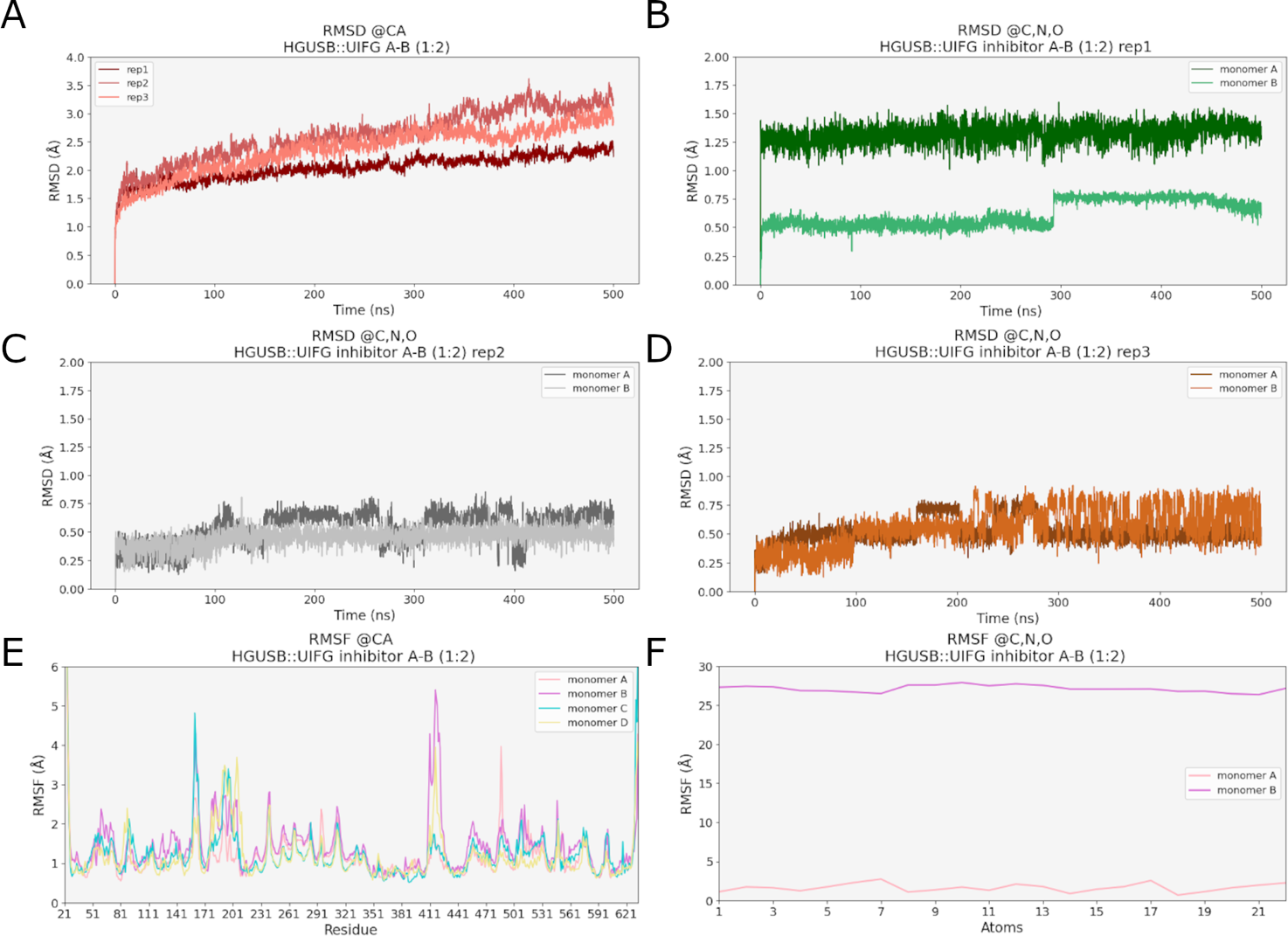


**Fig S7. RMSD and RMSF plots of the HGUSB::UIFG A-B (1:2) complex.** (A) The RMSD plot of the Cα reach a plateau during the MD simulations but at different value. (B) The RMSD profile of the inhibitor in the binding site, particularly for monomer A, reported values around 1.5 Å throughout the entire MD simulation in the first replicate. (C) The RMSD profile of the inhibitor in both monomers showed values around 0.5 Å during the MD simulation in the second replicate. (D) The RMSD profile of the inhibitor, in the last replicate, remained below 0.5 Å but exhibited more oscillations compared to the previously examined system. (E) The RMSF profile from the N- and C-termini indicated that all residues demonstrated RMSF values ≤ 6 Å, with fluctuations observed in regions similar to those in the *apo* protein as well as in other regions. The highest peaks were found in the regions including residues 141-211, 411-431, and 451-591. (F) The RMSF plot of the inhibitor, showing different fluctuations when bound to the two monomers, specifically reported a very high fluctuation value around 27 Å for the inhibitor in monomer B.


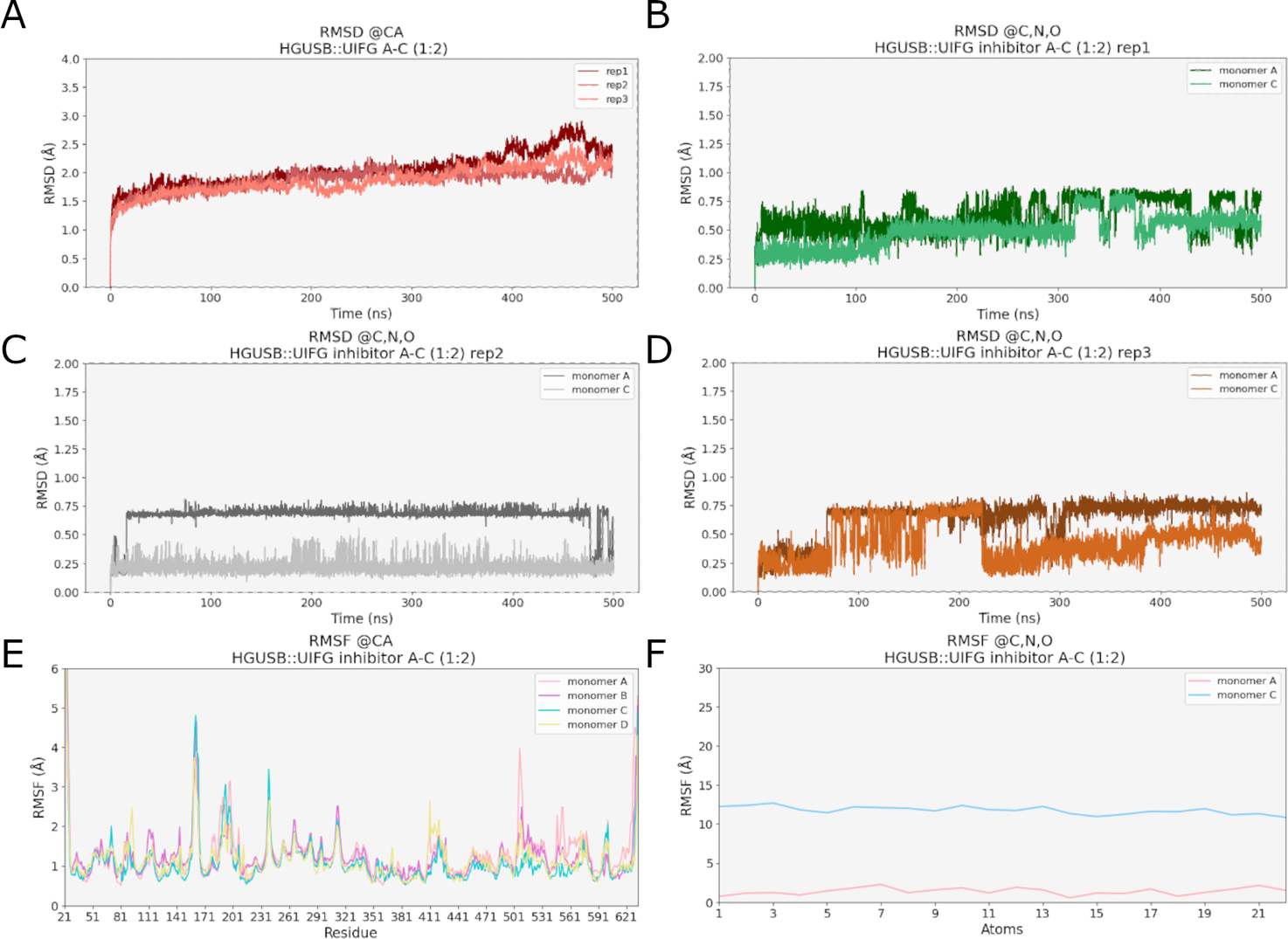


**Fig S8. RMSD and RMSF plots of the HGUSB::UIFG A-C (1:2) complex**. (A) The Cα RMSD plot reaches a plateau at 2.0 Å after 250 ns, with slight oscillations towards the end of the simulations. In the final 50 ns, the RMSD value stabilizes at 2.0 Å in all simulations. (B) The RMSD profile of the inhibitor in the binding site shows a value between 0.25 Å – 0.75 Å in the first replicate. (C) The RMSD profile of the inhibitor in both monomers displays a stable profile throughout the entire MD simulation in the second replicate. (D) The RMSD profile of the inhibitor reports a value around 0.25 Å – 0.75 Å for the entire MD simulation in the last replicate, with a stable profile after 250 ns. (E) The RMSF profile from the N- and C-termini shows all residues with RMSF values ≤ 5 Å, with small fluctuations in the same regions as the apo-protein, indicating a consistent trend across all monomers. (F) The RMSF plot of the inhibitor compares fluctuations when bound to monomers A and C. Specifically, for the inhibitor in monomer C, a fluctuation value around 12 Å is reported, but the inhibitor remains bound to the binding site.


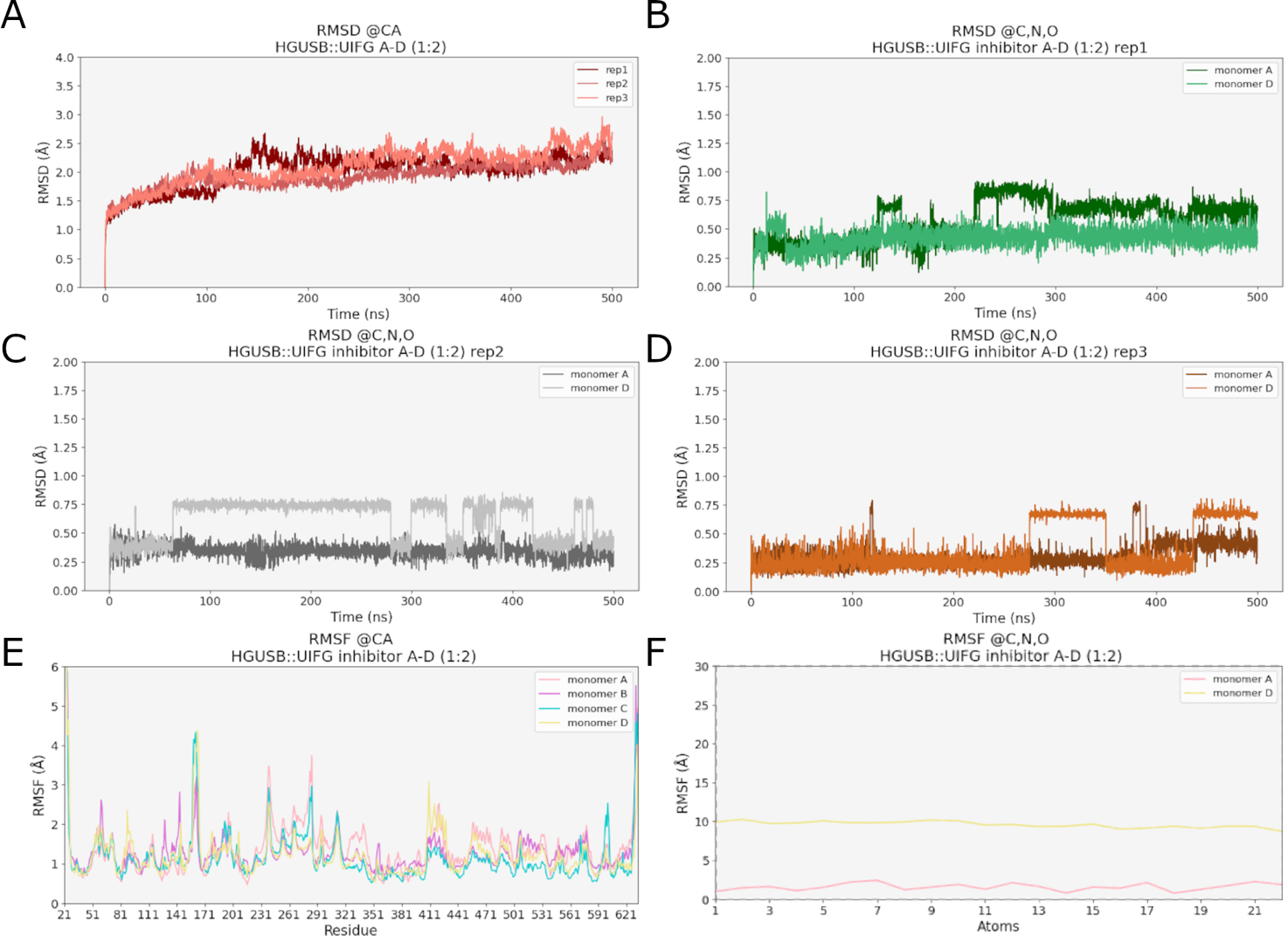


**Fig S9. RMSD and RMSF plot of the HGUSB::UIFG A-D (1:2).** (A)The Cα RMSD plot reaches a plateau after 250 ns, the average value during the simulation was about 2.5 Å. (B) RMSD profile of the inhibitor in the binding site, reporting value around 0.5 Å for the UIFG in the monomer D and about 1.0 Å for UIFG in the monomer A, in the first replica. (C) RMSD profile of the inhibitor in monomer A, reporting a stable profile for the entire MD simulation, while for the monomer D the value of RMSD fluctuates from 0.5 Å to 1.0 Å, in the second replica. (D) RMSD profile of the inhibitor, reporting value around 0.5 Å for the first 280 ns of MD simulation and value between 0.5 Å to 1.0 Å for the last 220 ns. (E) RMSF profile from the N- and C-termini, all residues demonstrate RMSF values ≤ 5 Å, with fluctuation in the same regions of the apo-protein. In particular, the RMSF profile the graph has a larger fluctuation in the residues 231-321. (F) UIFG RMSF profile in monomers A and D. In particular, for the inhibitor in monomer D, a value of 10 Å is reported, but the inhibitor remains bound to the binding site.


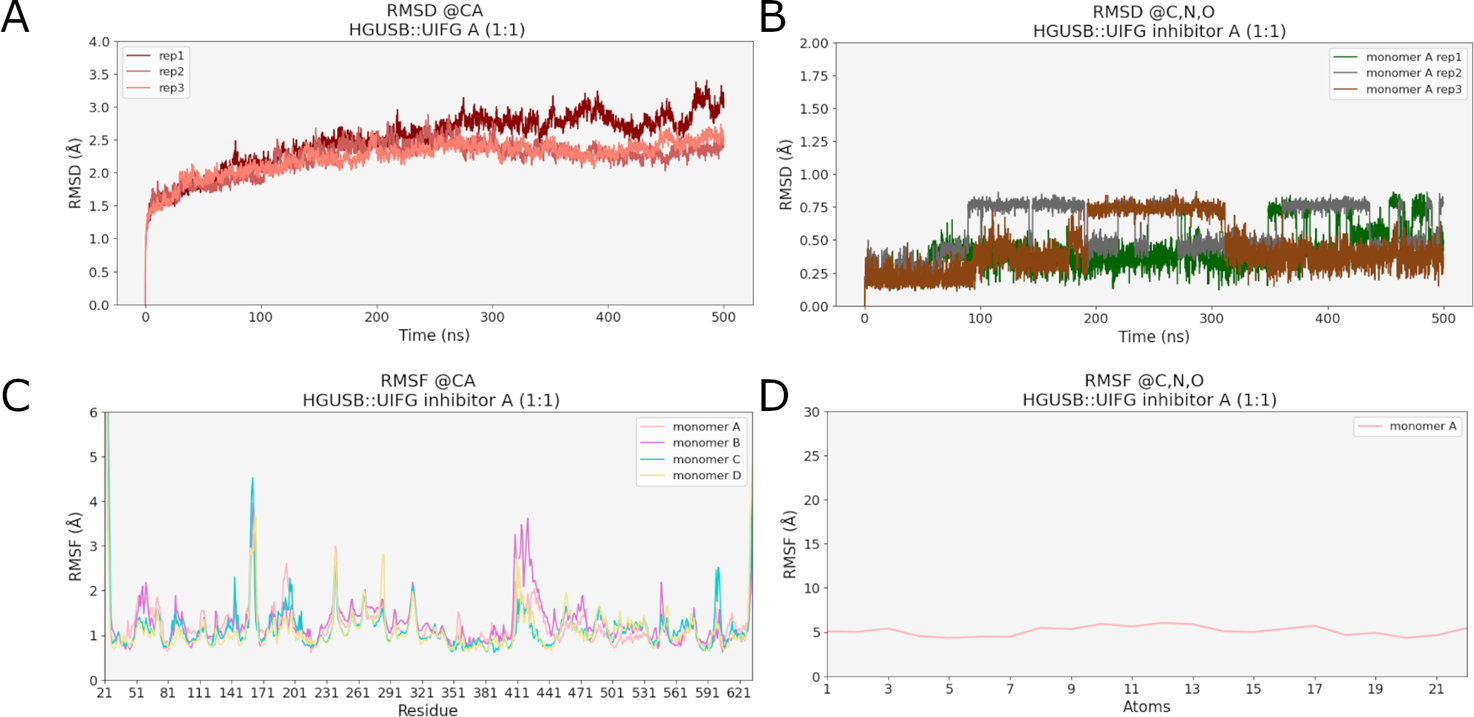


**Fig S10. RMSD and RMSF plots of the HGUSB::UIFG A (1:1).** (A) The Cα RMSD plot reaches a plateau after 250 ns, with an average value of approximately 2.5 Å during the simulation. (B) RMSD profile of the inhibitor in the binding site, showing values between 0 and 1 Å with fluctuations throughout the simulation. (C) Cα RMSF plot indicates fluctuations similar to the apo protein, but with higher values for residues between 411-441. (D) RMSF profile of the inhibitor in monomer A. This 1:1 configuration yields an RMSF value for the inhibitor higher than all previous experiments for the inhibitor that binds to monomer A.


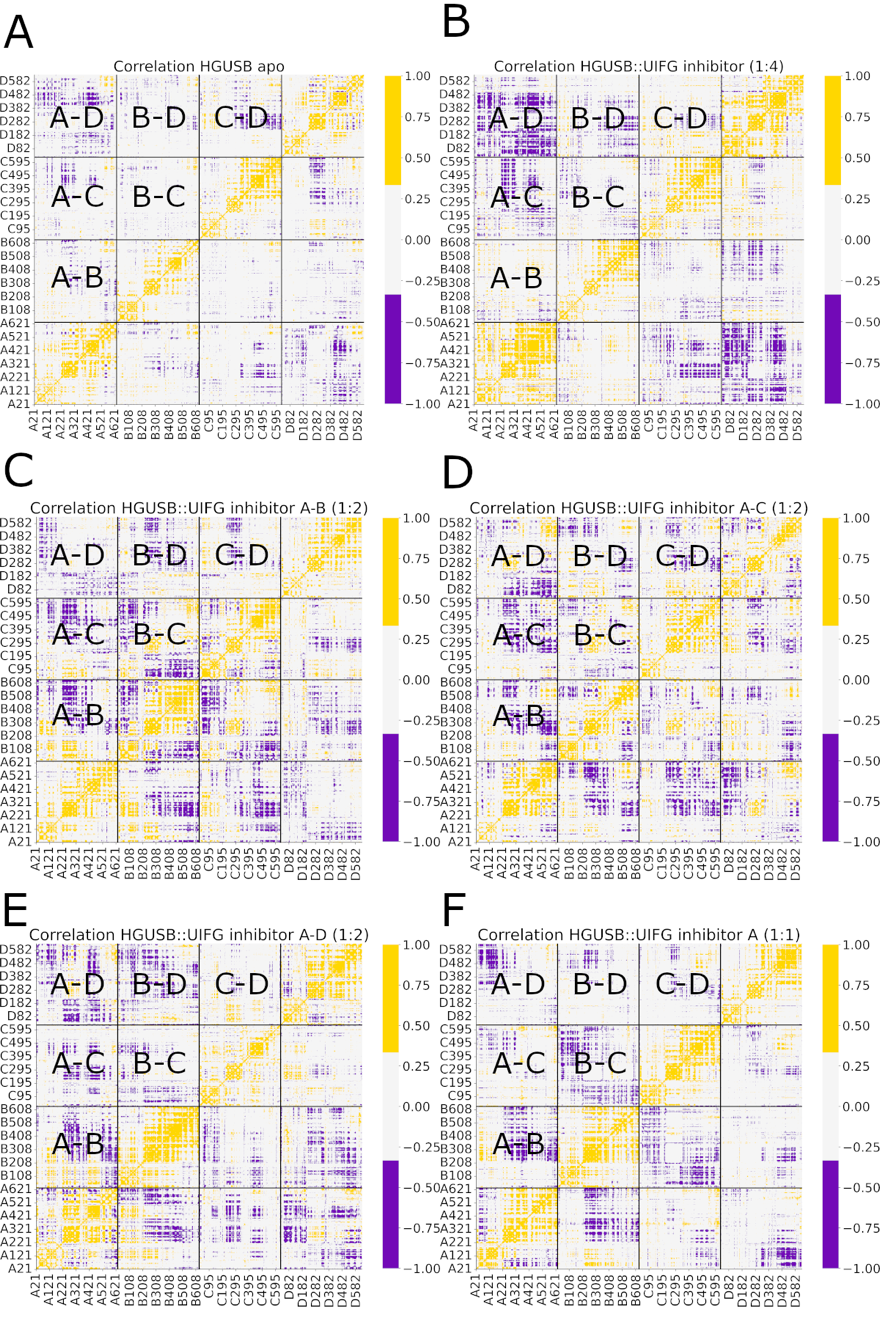


**FigS11.** DCC matrix of Cα atoms in all the simulated conditions. Yellow color code indicates positive correlations, whereas purple color code indicates negative correlations, for the extents of correlated and anti-correlated motions, respectively. (A) Correlation matrix of *apo*-protein. (B) Correlation matrix for HGUSB::UIFG inhibitor (1:4). (C) Correlation matrix for HGUSB::UIFG inhibitor A-B (1:2). (D) Correlation matrix for HGUSB::UIFG inhibitor A-C (1:2). (E) Correlation matrix for HGUSB::UIFG inhibitor A-D (1:2). (F) Correlation matrix for HGUSB::UIFG inhibitor A (1:1).


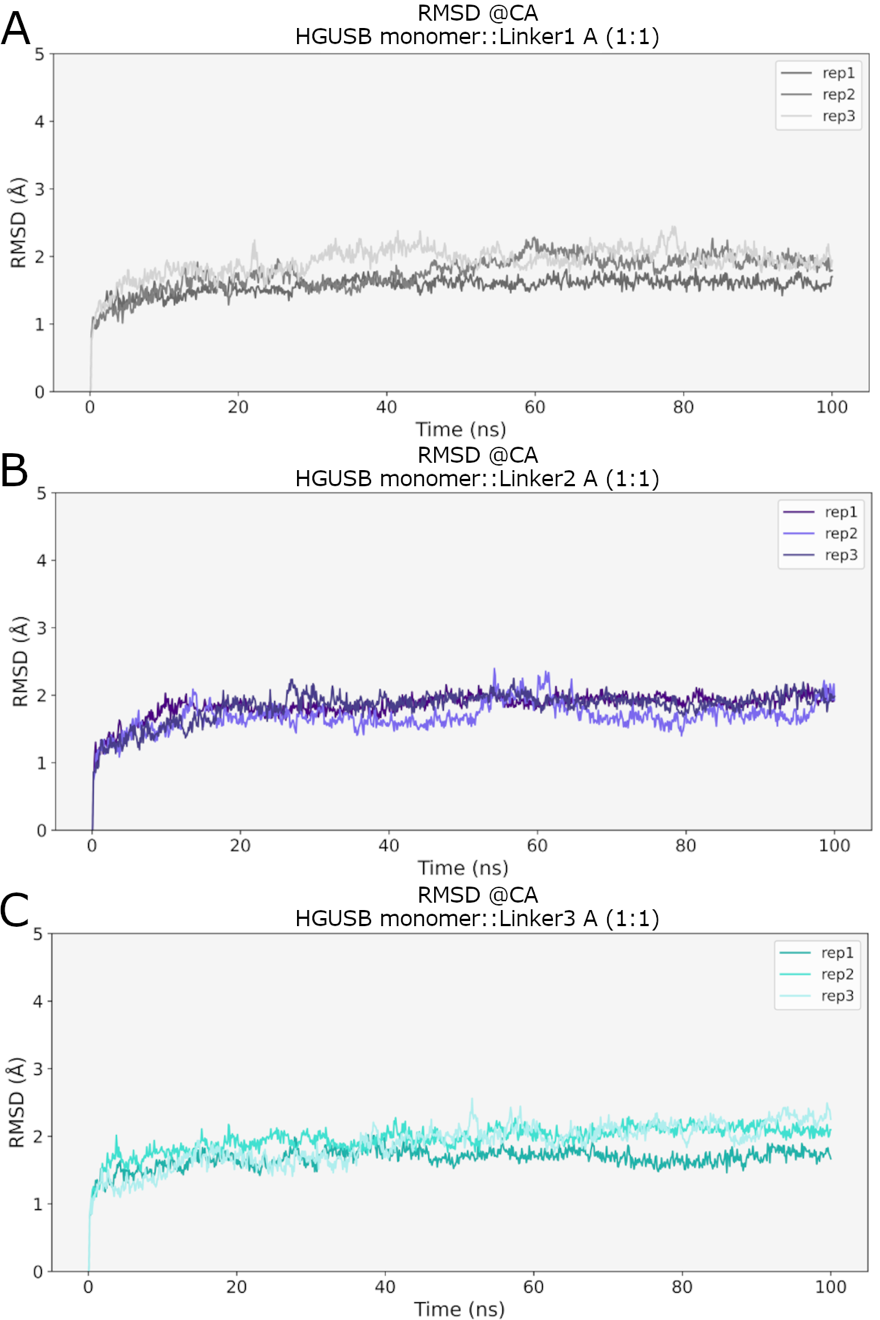


**Fig S12. Structural analysis of the HGUSB monomer::Linker systems, showing that in all cases Cα RMSD profile reaches a plateau at low values 2 Å.** (A) Cα RMSD profile for each HGUSB::Linker1 simulation. (B) Cα RMSD profile for each HGUSB::Linker2 simulation (C) Cα RMSD profile for each HGUSB::Linker3 simulation.


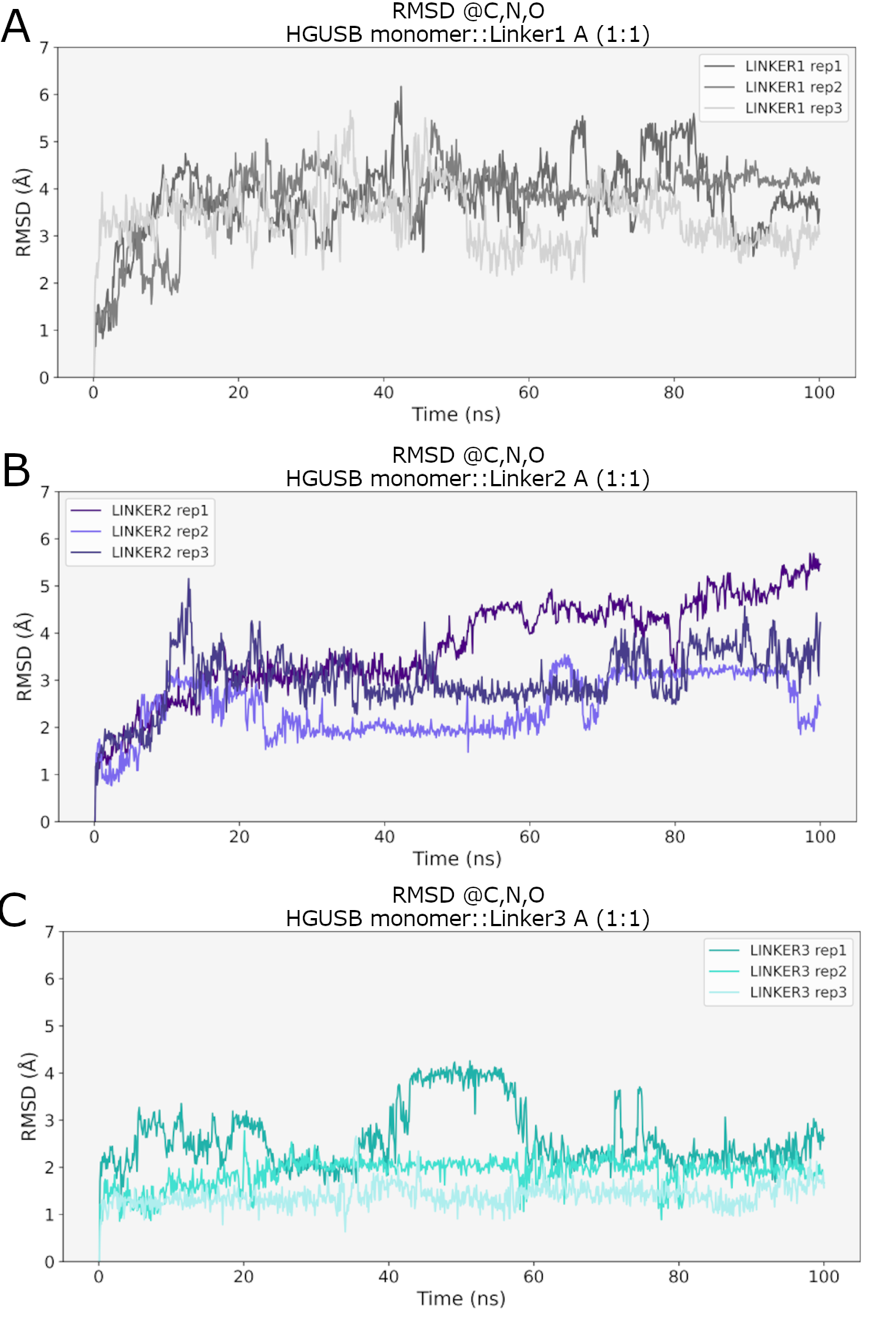


**Fig S13. RMSD profile of the linkers in all replicas for HGUSB monomer::Linker systems.** (A) RMSD profile of the Linker1. (B) RMSD profile of the Linker2. (C) RMSD profile of the Linker3.


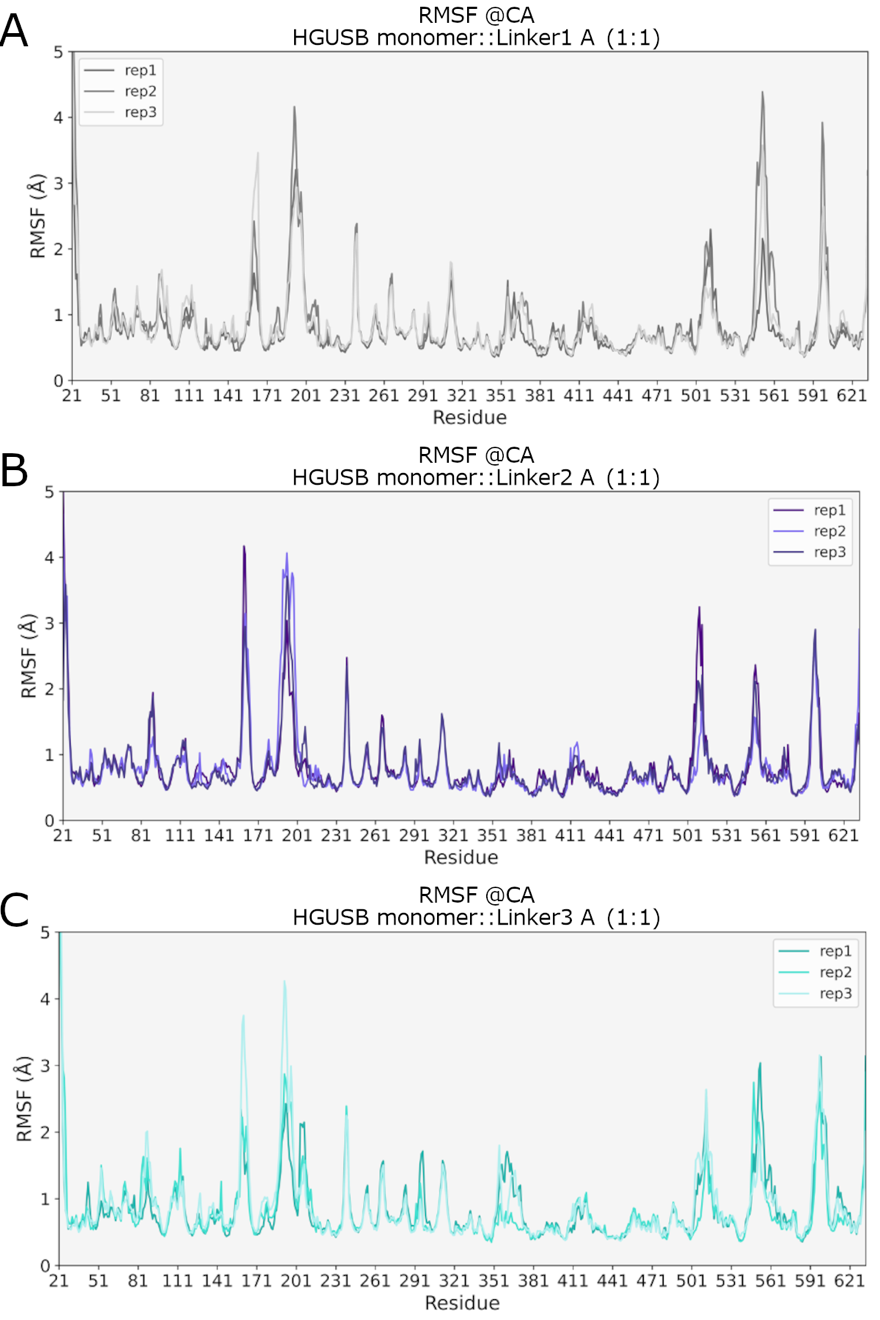


**Fig S14. Cα RMSF plots of the HGUSB monomer::Linker.** (A) RMSF plot HGUSB::Linker1. (B) RMSF plot HGUSB::Linker2. (C) RMSF plot HGUSB::Linker3.


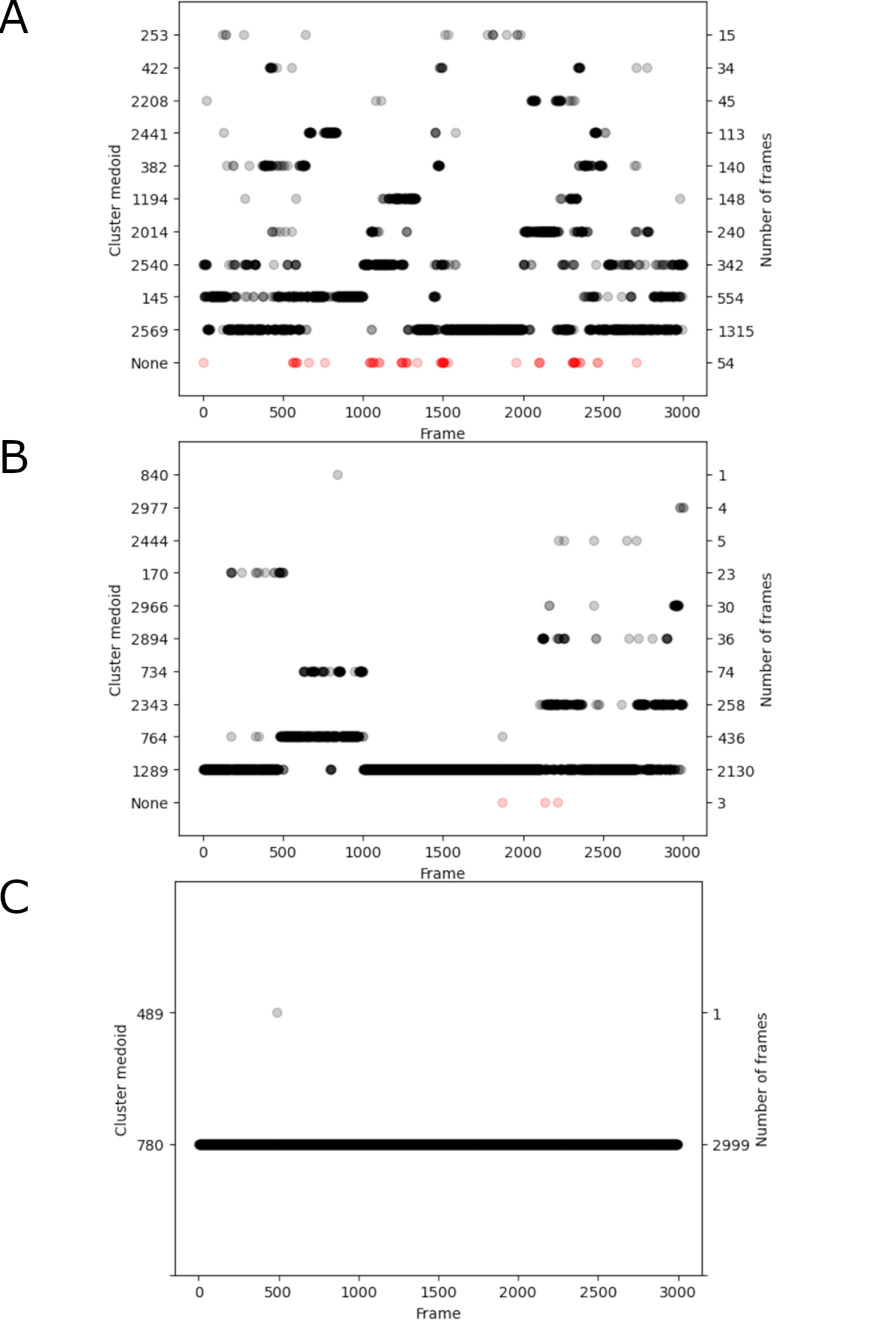


**Fig S15. Cluster Analysis.** (A) Medoid for Linker1. (B) Medoid corresponding to Linker2. (C) Medoid for Linker3.
